# Systematic analyses identify the anti-fibrotic role of lncRNA TP53TG1 in IPF

**DOI:** 10.1038/s41419-022-04975-7

**Published:** 2022-06-04

**Authors:** Jian Sun, Yingying Guo, Tingting Chen, Tongzhu Jin, Lu Ma, Liqiang Ai, Jiayu Guo, Zhihui Niu, Ruoxuan Yang, Qianqian Wang, Xiaojiang Yu, Huiying Gao, Yuhan Zhang, Wei Su, Xiaoying Song, Weihang Ji, Qing Zhang, Mengqin Huang, Xingxing Fan, Zhimin Du, Haihai Liang

**Affiliations:** 1 Zhuhai People’s Hospital, Guangdong Provincial Key Laboratory of Tumor Interventional Diagnosis and Treatment, Zhuhai Hospital Affiliated With Jinan University, Jinan University, Zhuhai, 519000 Guangdong China; 2 Department of Pharmacology (State-Province Key Laboratories of Biomedicine-Pharmaceutics of China, Key Laboratory of Cardiovascular Research, Ministry of Education), College of Pharmacy, Harbin Medical University, Harbin, 150081 China; 3 Department of Systems Biology, College of Bioinformatics Science and Technology, Harbin Medical University, Harbin, 150081 China; 4 State Key Laboratory of Quality Research in Chinese Medicine/Macau Institute for Applied Research in Medicine and Health, Macau University of Science and Technology, Macau (SAR), China; 5 Institute of Clinical Pharmacy, the 2nd Affiliated Hospital, Harbin Medical University, Harbin, 150081 China; 6 Research Unit of Noninfectious Chronic Diseases in Frigid Zone (2019RU070), Chinese Academy of Medical Sciences, Harbin, 150081 China

**Keywords:** Long non-coding RNAs, Respiratory tract diseases

## Abstract

Long non-coding RNA (lncRNA) was reported to be a critical regulator of cellular homeostasis, but poorly understood in idiopathic pulmonary fibrosis (IPF). Here, we systematically identified a crucial lncRNA, p53-induced long non-coding RNA TP53 target 1 (TP53TG1), which was the dysregulated hub gene in IPF regulatory network and one of the top degree genes and down-regulated in IPF-drived fibroblasts. Functional experiments revealed that overexpression of TP53TG1 attenuated the increased expression of fibronectin 1 (Fn1), Collagen 1α1, Collagen 3α1, ACTA2 mRNA, Fn1, and Collagen I protein level, excessive fibroblasts proliferation, migration and differentiation induced by TGF-β1 in MRC-5 as well as PMLFs. In vivo assays identified that forced expression of TP53TG1 by adeno-associated virus 5 (AAV5) not only prevented BLM-induced experimental fibrosis but also reversed established lung fibrosis in the murine model. Mechanistically, TP53TG1 was found to bind to amount of tight junction proteins. Importantly, we found that TP53TG1 binds to the Myosin Heavy Chain 9 (MYH9) to inhibit its protein expression and thus the MYH9-mediated activation of fibroblasts. Collectively, we identified the TP53TG1 as a master suppressor of fibroblast activation and IPF, which could be a potential hub for targeting treatment of the disease.

## Introduction

Idiopathic pulmonary fibrosis (IPF) is an irreversibly and devastating lung parenchymal fibrosis with unknown etiology [1]. Clinical data from the COVID-19 pandemic also indicate that pulmonary fibrosis may occur after infection that affects the outcome of patients [2]. As no drug to date has been fully clinically effective in halting fibrosis progression, it is imperative to reveal the landscape of potential fibrotic effectors and the molecular mechanisms by which they initiate and amplify IPF.

Currently, it is believed that IPF develops as a result of the interactions between complex intracellular signaling cascades and multiple cell types, including immune cells, epithelial cells, and fibroblasts, bringing forth the importance of cell heterogeneity [3]. Two approved drugs for the treatment of IPF, Pirfenidone, and Nintedanib, partly delay or attenuate the progression of IPF by inhibiting myofibroblast differentiation [4, 5], highlight the intraalveolar fibroblasts activation and the subsequent accumulation of extracellular matrix proteins as a classic pathological progression of IPF [6]. Understanding the regulatory mechanisms controlling lung fibroblast activation is essential to develop novel therapeutic strategies.

Long non-coding RNAs (LncRNAs), as regulators of a variety of biological processes, have been shown to be involved in the pathophysiology of IPF, including fibroblast activation [7]. Our previous study suggested that lncRNA PFAR, identified with the mouse fibrosis arrays, promotes fibroblast differentiation during pulmonary fibrosis by sponging miR-138 to regulate the YAP1-Twist axis [8]. However, lncRNA has been reported to show poor conservation of sequences among species, limiting practical usage and further clinical application. Savary et al. identified lncRNA DNM3OS by mouse and human microarray, as a fibroblast-specific critical effector of TGF-β-induced fibroblast activation. It regulated the fibrogenic process by giving rise to three distinct profibrotic mature miRNAs (miR-199a-5p/3p and miR-214-3p), which affect SMAD and non-SMAD components of TGF-β signaling [9]. Therefore, we focused on exploring the driver lncRNA differentially expressed in IPF patients and revealing the underlying molecular mechanism.

The crosstalk between mRNAs and lncRNAs indeed provided significant insights into the regulatory mechanism underlying pulmonary fibrosis [10]. Li et al. revealed a pulmonary fibrosis-related genes crosstalk of differentially expressed mRNA and non-coding RNA (ncRNA) that obtained by RNA sequencing in bleomycin (BLM)-induced mice, leading to a new treatment for pulmonary fibrosis [11]. Our previous study constructed a IPF progression-related lncRNAs-mRNAs co-expression networks, revealed a key lncRNA CTD-2528L19.6, which was up-regulated in early-stage IPF and subsequently down-regulated during advanced-stage IPF patients [12]. Thus, the construction of a gene regulatory network to provide new perspectives for evaluating key regulators in IPF has become an essential strategy to reveal the mechanism of diseases.

In this study, we globally profiled lncRNA expression in IPF and found an important gene hub gene tumor protein 53 target gene 1 (TP53TG1). Nevertheless, the biological function and detailed molecular mechanisms of TP53TG1 remain to be elucidated in pulmonary fibrosis. TP53TG1 exhibited distinct expression patterns in diverse cell types determined by analyzing single-cell RNA sequencing (scRNA-seq) dataset and downregulated in IPF-derived fibroblast. We hypothesized that TP53TG1 participates in the development of IPF by regulating the fibrogenesis of fibroblasts, and explored its mechanism.

## Results

### Analysis of IPF related co-expression network

As summarized in Supplementary Fig. 1, we firstly performed two groups of comparisons: (1) early IPF lung vs. normal lung, (2) advanced IPF lung vs. normal lung, in whole lung samples from GSE24206. 48 lncRNAs and 783 mRNAs were significantly differentially expressed in both comparisons (*P*-value < 0.01, Student’s *t*-test). Meanwhile, in IPF lung fibroblasts samples from GSE44723, we identified 12 lncRNAs and 351 mRNAs that were significantly differentially expressed for the comparison of IPF fibroblasts vs. normal fibroblasts (*P*-value < 0.01, Student’s *t*-test). These four groups of genes were defined as IPF-related genes.

**Fig. 1:**
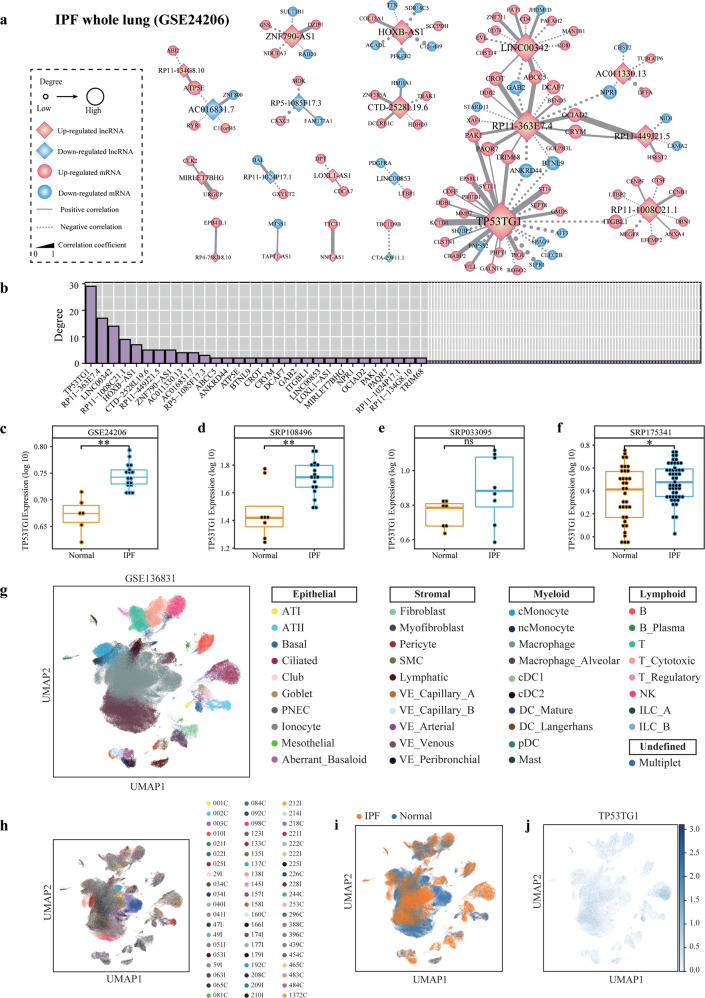
LncRNA-mRNA co-expression networks in whole lung samples of IPF. **a** IPF-related lncRNAs-mRNAs co-expression network in GSE24206. The diamond and round nodes are lncRNAs and mRNAs, respectively. Nodes marked with pink color represent the up-regulated IPF related genes. Nodes marked with blue color represent the down-regulated IPF-related genes. The size of nodes represents the degree and the line thickness of the edges indicates the strength of correlation. The solid lines (dotted lines) represent the positive (negative) correlation between mRNAs and lncRNAs (|r| > 0.8, *P*-value < 0.01, Pearson Correlation Test). **b** Distribution of the degree of genes in the IPF-related network (genes with degree greater than two were shown). TP53TG1 was the hub gene. **c**–**f** The expression of TP53TG1 in IPF compared to normal lung in four independent datasets (GSE24206, SRP108496, SRP033095 and SRP175341). *P-value < 0.05, **P-value < 0.01 in Student’s *t*-test. “ns”, no significance. **g** UMAP representation of 243,472 cells from 32 IPF and 28 normal donor lungs. Each dot represents a single cell, and cells are labeled as one of 39 discrete cell varieties. UMAP plot of all the single cells, with each color coded for (**h)** 60 patients, (**i)** sample origin (Normal or IPF). **j** Expression of TP53TG1.

To capture the core lncRNA-mRNA regulatory module over pathogenesis in IPF, we constructed an IPF pathogenic co-expression network in IPF whole lungs (Fig. 1a). A subnetwork of 29 genes centered on the hub gene TP53TG1 (Fig. 1b), and the expression of TP53TG1 was consistently up-regulated in four independent datasets (GSE24206, SRP108496, SRP033095, and SRP175341) (Fig. 1c–f). Considering the heterogeneity in cellular microenvironment, we performed scRNA-seq analysis of more than 300,000 cells isolated from IPF and normal lungs. Unsupervised graph-based clustering identified 39 discrete cell clusters labeled by patient information, visualized with the Uniform Manifold Approximation and Projection (UMAP) algorithm (Fig. 1g–i). Based on relatively similar cell types, we grouped cells into four groups: epithelial, stromal, myeloid, and lymphoid. We found low expression of TP53TG1 in fibroblasts cells (stromal group) and high expression of TP53TG1 in ciliated, ATI cells (epithelial group) and basal cells (stromal group). In summary, IPF showed high heterogeneity and TP53TG1 exhibited distinct patterns of expression in different cell types (Fig. 1j).

### Enhanced expression of TP53TG1 prevents experimental lung fibrosis in mice

**Fig. 2:**
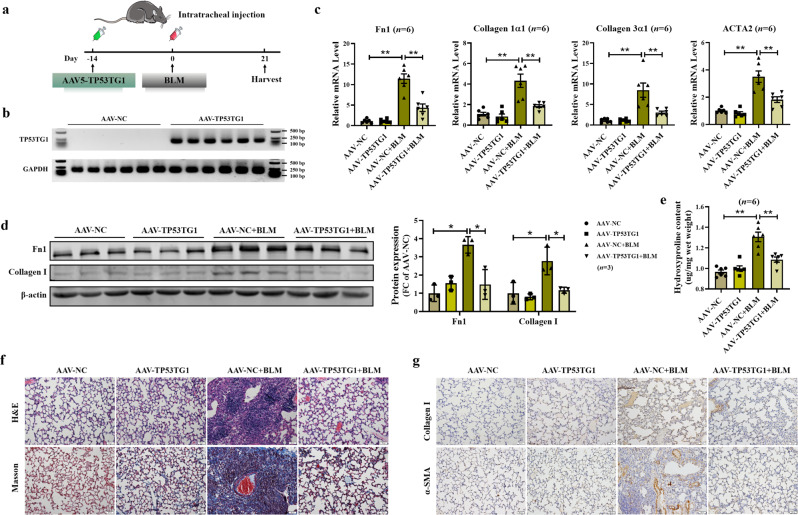
BLM-induced lung fibrosis is suppressed by preventive TP53TG1. **a** Diagram of the animal experimental mode. Mice received intratracheal injection of AAV5-TP53TG1 14 days before BLM induction, and lung tissues were harvested 21 days later. **b** TP53TG1 was significantly overexpressed in lung of AAV5-TP53TG1 infected mice confirmed by agarose gel electrophoresis; *n* = 6. **c** The relative expression of fibrosis related genes induced by BLM was suppressed by prior injection of AAV5-TP53TG1. **d** The effect of TP53TG1 on the protein levels of Fn1 and Collagen I in vivo was assessed by Western blots. **e** Hydroxyproline content was significantly reduced in the lungs of AAV5-TP53TG1 treated mice. **f** H&E and Masson staining indicated that TP53TG1 inhibited BLM-induced lung morphological changes and increased fibrotic area; *n* = 3. **g** Immunohistochemical experiments detected lighter staining for Collagen I and α-SMA in the AAV5-TP53TG1-treated BLM-mice; *n* = 3. *P < 0.05; **P < 0.01. BLM, bleomycin; AAV-TP53TG1, adeno-associated virus 5 containing TP53TG1; FC fold change.

Since we have found the TP53TG1 expression in IPF patients, we next explored the potential roles of TP53TG1 in experimental lung fibrosis in mice. We generated adeno-associated virus 5 (AAV5) containing TP53TG1 overexpression plasmid and intratracheally injected two weeks before administration of BLM, and the mice were euthanized after three weeks (Fig. 2a, b). According to qRT-PCR analysis, the mRNA levels of Fn1, Collagen 1α1, Collagen 3α1, and ACTA2 of mice challenged with BLM were suppressed by AAV5-TP53TG1, whereas AAV5-TP53TG1 alone had no effect on fibrosis-related mRNA levels (Fig. 2c). Western blots analysis indicated that Fn1 and Collagen I induced by BLM were impeded by AAV5-TP53TG1 (Fig. 2d). Hydroxyproline content in lung tissues of mice treated with AAV5-TP53TG1 was lower compared with that in BLM-treated mice (Fig. 2e). The BLM-instilled mice injected with AAV5-TP53TG1 in advance showed noticeable improvement in lung tissue morphology and fibrotic area by H&E and Masson staining, indicating that TP53TG1 inhibited fibrogenesis in vivo (Fig. 2f). Additionally, we confirmed our findings by immunohistochemistry. The increased expression of Collagen I and α-SMA induced by BLM was significantly inhibited by AAV5-TP53TG1 (Fig. 2g). These results illustrate that TP53TG1 can play a protective role in BLM-induced pulmonary fibrosis as a means of prevention. Our unexpected findings prompted us to thoroughly investigate the anti-fibrotic potential of TP53TG1.

### Related co-expression network in IPF fibroblasts

**Fig. 3:**
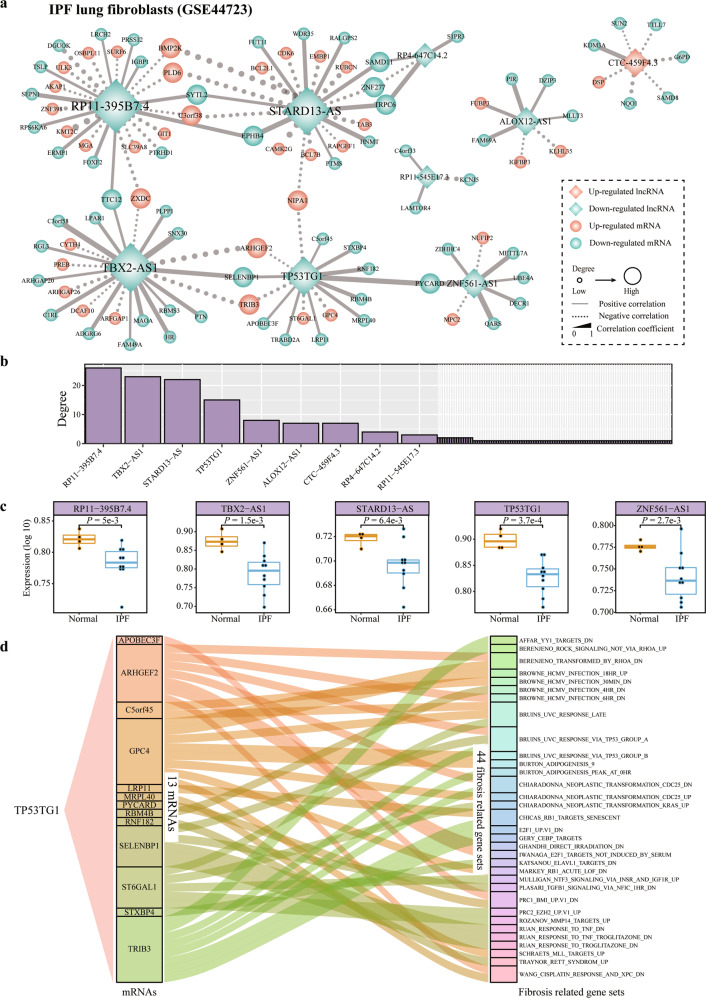
LncRNA-mRNA co-expression networks in lung fibroblasts samples from IPF. **a** IPF-related lncRNAs-mRNAs co-expression network in GSE44723. The diamond and round nodes are lncRNAs and mRNAs, respectively. Nodes marked with orange color represent the up-regulated IPF-related genes. The nodes marked with green color represent the down-regulated IPF-related genes. The size of nodes represents the degree and the line thickness of the edges indicates the strength of correlation. The solid lines (dotted lines) represent the positive (negative) correlation between mRNAs and lncRNAs (|r| > 0.8, *P*-value < 0.01, Pearson Correlation Test). **b** Distribution of the degree of genes in the IPF-related network (genes with degree greater than three were shown). **c** The expression of the five genes in IPF compared to normal lung. The Student’s *t*-test was used to determine the statistically significant differences between two groups. **d** Sankey diagrams display the connection between TP53TG1 correlated mRNAs and fibrosis-related gene sets in IPF.

The expression of TP53TG1 displayed an additional layer of complexity in cell-type composition. The heterogeneity in cells may explain the undefined function of TP53TG1 in whole lung models of animals. This hypothesis inspired us to construct an IPF co-expression network in a typical population of cells: lung fibroblasts (Fig. 3a). LncRNA RP11−395B7.4, TBX2−AS1, STARD13−AS, TP53TG1, and ZNF561−AS1 with top degree were significantly down-regulated in IPF fibroblasts compared with normal fibroblasts **(***P*-value < 0.05, Student’s *t*-test, Fig. 3b). Among the five lncRNAs, the expression of TP53TG1 showed the greatest difference between IPF and normal lungs (Fig. 3c). TP53TG1 may mediate the function of fibrogenesis by regulating genes ARHGEF2, GPC4, and TRIB3 et al. in fibrosis-related genesets (Fig. 3d).

### Overexpression of TP53TG1 significantly inhibits the fibrogenesis of MRC-5/PMLFs induced by TGF-β1

**Fig. 4:**
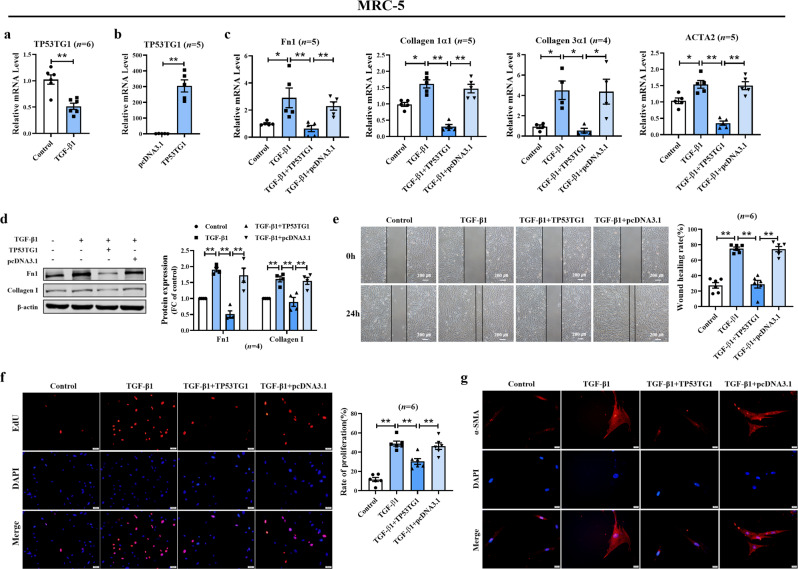
Effects of TP53TG1 on human lung fibroblasts (MRC-5). **a** After TGF-β1 induction, TP53TG1 was down-regulated measured by qRT-PCR. **b** TP53TG1 plasmid overexpression efficiency was verified by qRT-PCR. **c** The suppression of relative mRNA expression of fibrosis markers by TP53TG1 was examined by qRT-PCR. **d** TP53TG1 showed noticeable inhibitory effects on TGF-β1-induced upregulation of Fn1 and Collagen I protein levels. **e**, **f** TP53TG1 hindered the migration and proliferation of MRC-5 cells induced by TGF-β1 detected by wound healing (bar = 200 μm) and EdU experiments (bar = 50 μm). **g** Immunofluorescence analysis revealed that TP53TG1 regulated the generation of α-SMA positive cells in human fetal lung fibroblasts (bar = 20 μm; *n* = 5). *P < 0.05, **P < 0.01. FC fold change.

To investigate the functional role of TP53TG1 in fibroblasts, we performed loss- or gain-of-function experiments. Firstly, we observed a statistically significant reduction of TP53TG1 in TGF-β1 induced MRC-5 cells (Fig. 4a), which was consistent with the results in IPF-drived fibroblasts. Then, we used a smart silencer to knock down TP53TG1 and confirmed the depletion efficiency in MRC-5 cell line (Supplementary Fig. 2a). The extracellular matrix protein Fn1, Collagen 1α1, TIMP1 was substantially altered in TP53TG1 knockdown cells (Supplementary Fig. 2b). Moreover, silencing of TP53TG1 promoted MRC-5 proliferation and differentiation, which indicates that TP53TG1 silencing induces fibrogenesis of MRC-5 (Supplementary Fig. 2c, d).

To further understand whether TP53TG1 produces a marked effect through fibroblasts, we constructed TP53TG1 overexpression plasmids to enhance TP53TG1 expression in MRC-5 successfully (Fig. 4b). The mRNA expression of Fn1, Collagen 1α1, Collagen 3α1 and ACTA2 were up-regulated in MRC-5 cells induced by TGF-β1, but these alterations were reversed after TP53TG1 overexpression (Fig. 4c). In addition, the protein expression of Fn1 and Collagen I were lower in TP53TG1 transfected MRC-5 cells than that in TGF-β1-induced cells (Fig. 4d). Since the overactivation of fibroblasts is an essential feature of pulmonary fibrosis, we evaluated the regulatory effects of TP53TG1 on the function of MRC-5 cells. The results of the wound-healing assay showed that TP53TG1 inhibited the migration ability of lung fibroblasts (Fig. 4e). Furthermore, TP53TG1 was confirmed to reduce the increased cell proliferation induced by TGF-β1 according to EdU fluorescence staining (Fig. 4f). Immunofluorescence staining also showed that the transition of fibroblasts into α-SMA positive myofibroblasts was diminished in the presence of TP53TG1 (Fig. 4g). Our results suggest that TP53TG1 attenuates TGF-β1 induced fibrogenesis in MRC-5 cells, and the prevention of experimental pulmonary fibrosis in mice may be due to this function.

**Fig. 5:**
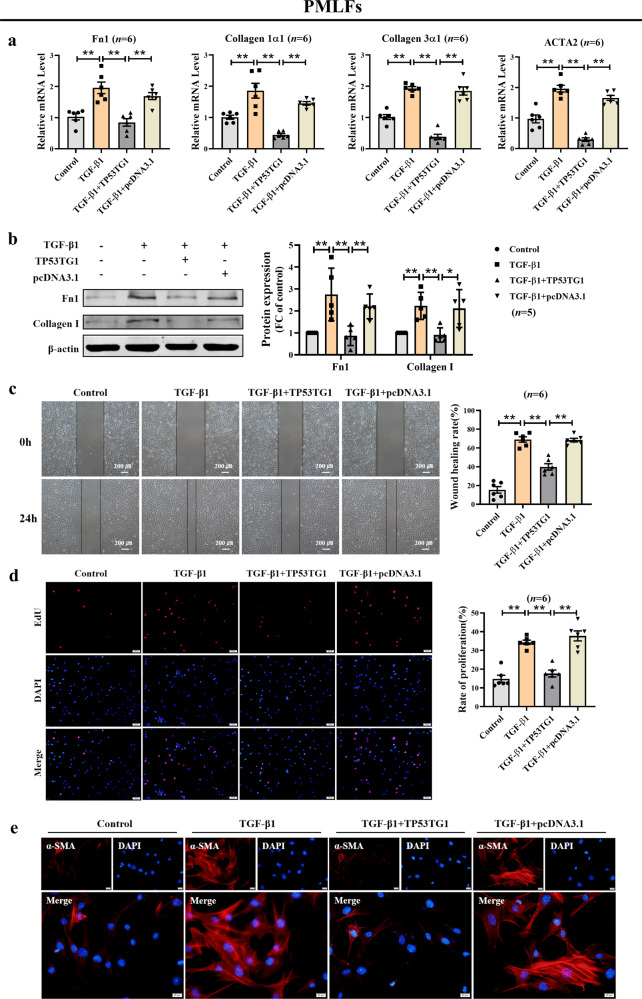
Anti-fibrotic effects of TP53TG1 in PMLFs. **a** The relative mRNA levels of Fn1, Collagen 1α1, Collagen 3α1, and ACTA2 in TP53TG1 transfected cells after TGF-β1 induction. **b** TP53TG1 inhibited the protein expression of fibrotic markers caused by TGF-β1 in PMLFs. **c** The migratory of PMLFs was attenuated by TP53TG1, as detected by the wound healing assay. (bar = 200 μm). **d** The fluorescence results of the EdU assay showed the cell proliferation of PMLFs. (bar = 50 μm). **e** Immunofluorescence staining indicated that TGF-β1-induced α-SMA positive cells, which were impeded by the enhanced expression of TP53TG1 in PMLFs. (bar = 20 μm; *n* = 5). *P < 0.05, **P < 0.01. PMLFs, primary mouse lung fibroblasts; FC fold change.

Consistent with the results observed in MRC-5 cells, forced expression of TP53TG1 inhibits the excessive proliferation, migration, differentiation, and ECM deposition induced by TGF-β1 in primary mouse lung fibroblasts (PMLFs) (Fig. 5a–e).

### Forced expression of TP53TG1 attenuates lung fibrosis progression in an established experimental model of lung fibrosis in mice

**Fig. 6:**
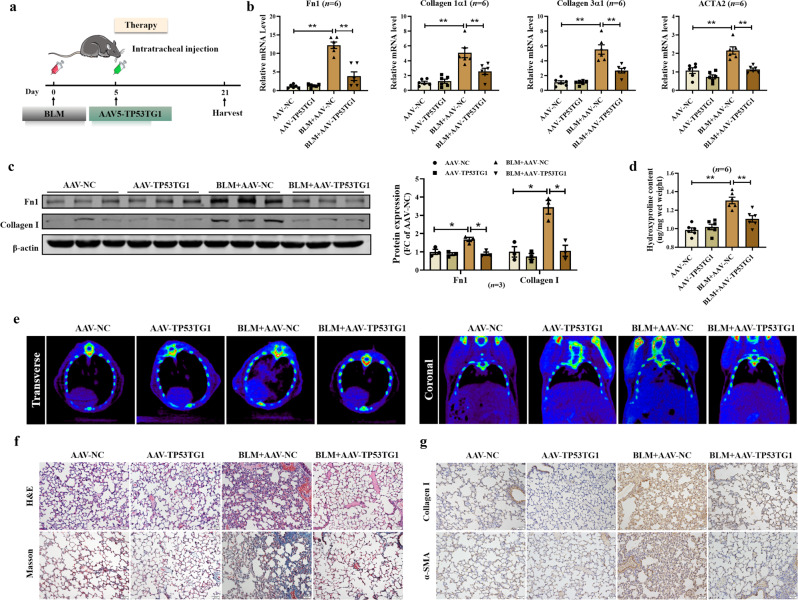
Inhibitory effect of therapeutic TP53TG1 on pulmonary fibrosis. **a** Diagram of the experimental animal model of therapeutic TP53TG1. Mice were intratracheally injected with AAV-TP53TG1 at day five after induction by BLM and sacrificed 21 days later. **b** Fibrosis markers mRNA level of Fn1, Collagen 1α1, Collagen 3α1, and ACTA2 in lungs after BLM/TP53TG1 treatment. **c** After fibrosis formation, TP53TG1 inhibited the relative protein expression of Fn1 and Collagen I. **d** BLM increased hydroxyproline content, which was reversed upon AAV5-TP53TG1 treatment. **e** CT scans of foci in the lung of mice; *n* = 3. **f** Therapeutic TP53TG1 alleviated BLM-induced fibrotic changes in lung tissue detected by H&E and Masson staining; *n* = 3. **g** Immunohistochemical staining showed that AAV5-TP53TG1 regulated the expression of Collagen I and α-SMA in lung tissues; *n* = 3. *P < 0.05; **P < 0.01. AAV-TP53TG1, adeno-associated virus 5 containing TP53TG1; FC fold change.

To assess the potential therapeutic benefit of TP53TG1 on BLM models that were already established, we intratracheal injected AAV5-TP53TG1 five days after BLM induction and euthanized the mice 21 days after BLM injection (Fig. 6a). After BLM successfully induced pulmonary fibrosis in mice, the mRNA levels of fibrosis-related genes were inhibited by the AAV5-TP53TG1 (Fig. 6b). And the protein expressions of Fn1 and Collagen I was similarly down-regulated by TP53TG1 treatment in BLM mice (Fig. 6c). AAV5-TP53TG1 treatment significantly attenuated the increased levels of hydroxyproline compared to the mice exposed to BLM (Fig. 6d). The chest CT of BLM-induced mice showed shadows, which was attenuated by AAV5-TP53TG1 (Fig. 6e). In response to therapeutic AAV5-TP53TG1, the lung tissues of mice showed less fibrotic area, lighter Collagen I and α-SMA staining (Fig. 6f–g), indicating that TP53TG1 is able to impede the progression of lung fibrosis.

### TP53TG1 directly binds to and represses MYH9 protein thereby alleviating fibroblast activation

**Fig. 7:**
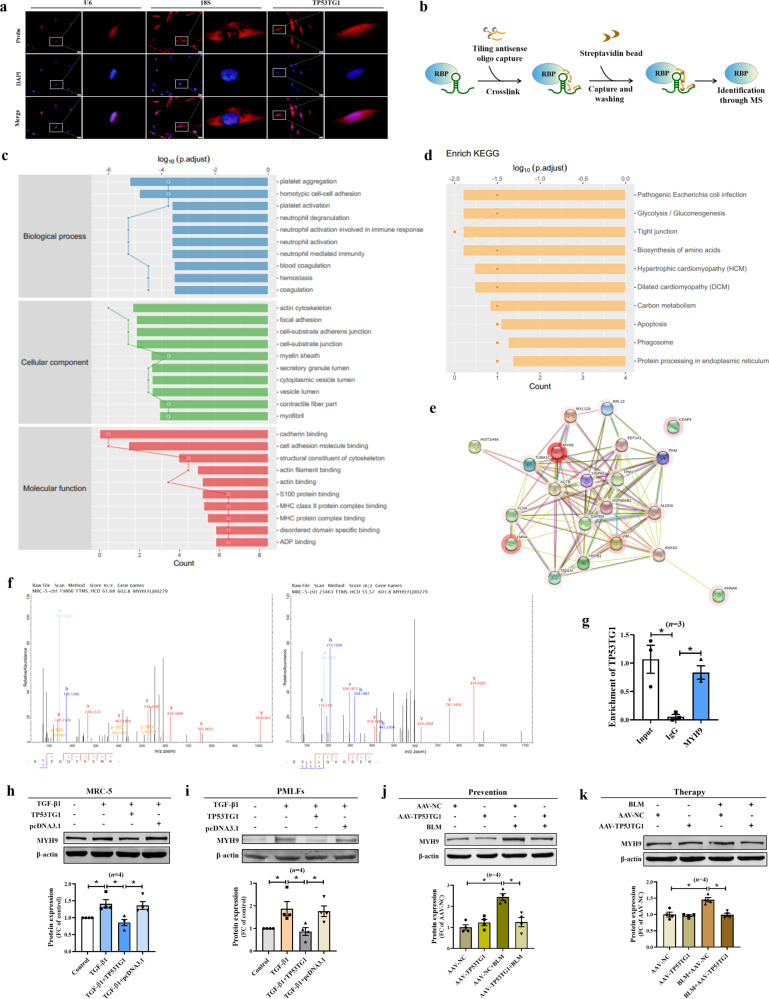
TP53TG1 regulates MYH9 expression through binding to it. **a** The FISH probe detected that TP53TG1 was expressed in the cytosol and nucleus in MRC-5; bar = 20 μm; *n* = 4. **b** Schematic diagram of ChIRP-MS. **c**, **d** KEGG and GO analysis of the TP53TG1 interactome demonstrated the enrichment of splicing factors among TP53TG1-interacting proteins. **e** The interaction network of TP53TG1 protein interactome. **f** The protein mass spectrum suggested the MYH9 protein may bind to the TP53TG1. **g** RNA-binding protein immunoprecipitation (RIP) confirmed the interactions between TP53TG1 and MYH9 in vitro. **h** TP53TG1 derepressed the MYH9 protein in MRC-5. **i** In PMLFs, the expression of MYH9 was reduced by TP53TG1 overexpression. **j**, **k** The protein level of MYH9 in lung tissue of AAV-TP53TG1 mice was significantly inhibited than that of BLM-treated mice. *P < 0.05. AAV-TP53TG1, adeno-associated virus 5 containing TP53TG1; FC fold change.

The specific regulatory mechanism of a lncRNA is closely related to its cellular localization. Through FISH experiments, we found that TP53TG1 was expressed in both the cytoplasm and the nucleus (Fig. 7a). It has been found that the primary mechanism by which lncRNAs participate in the regulation of biological processes in the nucleus depends on their binding to proteins and thus regulating their expression and activity. Therefore, we performed a comprehensive identification of RNA-binding proteins by mass spectrometry (ChIRP-MS) assay to explore the proteins bound by TP53TG1 and mediating its anti-fibrotic effects (Fig. 7b). The functional annotations of the interacting proteins were obtained from the Gene Ontology (GO) database, and pathway enrichment analysis was carried out using the Kyoto Encyclopedia of Genes and Genomes (KEGG) database. These analyses showed that the interacting proteome of TP53TG1 was significantly enriched in tight junction (Fig. 7c–e). To examine the function of TP53TG1 in IPF lung fibroblasts, we integrated co-expression, transcriptional regulation and PPI that linked to TP53TG1 (Supplementary Fig. 3a). Genes that correlated with TP53TG1 in the network were significantly enriched in transcriptional regulation, metabolism, immune related signaling pathways, epithelial cell function and cellular states related pathways, such as “Adherens junction”, “Cellular senescence”, “Mitophagy” (*FDR* < 0.05, Hypergeometric test, Supplementary Fig. 3b). Thus, TP53TG1 may be involved in IPF by regulating cell junction.

In particular, the mass spectrum assay suggested the MYH9 protein binds to TP53TG1 (Fig. 7f). In order to further confirm such binding, we performed RNA immunoprecipitation (RIP) assay in MRC-5. The results showed the physical interaction of TP53TG1 with MYH9 (Fig. 7g). Furthermore, western blot assays demonstrated overexpression of TP53TG1 inhibited TGF-β1-induced up-regulation of MYH9 in MRC-5/PMLFs (Fig. 7h–i). Consistent with these results, BLM administration promoted the expression of MYH9, whereas overexpression of TP53TG1 repressed the protein expression of MYH9 (Fig. 7j–k). These results suggest a direct interaction between MYH9 protein and TP53TG1, which may be involved in the pathological process of pulmonary fibrosis.

## Discussion

In this study, we constructed lncRNA-mRNA co-expression network and identified a key lncRNA TP53TG1 associated with IPF and IPF-fibroblast, and comprehensively analyzed the intercellular heterogeneity of TP53TG1. Prior exposure to AAV5-TP53TG1 attenuates BLM-induced lung fibrosis in mice. To explore the certain reason of our results, further bioinformatic analysis shows that TP53TG1 is substantially downregulated in the fibroblasts of IPF patients. We find enforced TP53TG1 expression represses TGF-β1-stimulated lung fibroblast-to-myofibroblast transition and attenuates established BLM induced lung fibrosis of mice.

Screening for novel lncRNAs is crucial to understand the biological functions of IPF. Here, we identified differentially expressed genes (DEGs) crosstalk between mRNAs and lncRNAs to gain insight into the regulatory mechanism in pulmonary fibrosis. Comprehensively analysis of lncRNA expression in IPF whole lung revealed several key candidate lncRNAs like TP53TG1, LINC00342, RP11-1008C21.1, HOXB-AS1, CTD-2528L19.6 et al., that were differentially overexpressed in IPF compared to both normal samples. LINC00342 was determined as a poor prognostic biomarker of non-small cell lung cancer positively correlated with lymph node metastasis and TNM stages [13, 14]. Bi et al. indicated HOXB-AS1 promotes HOXB2 or HOXB3 expression at the transcriptional and posttranscriptional levels in glioblastoma to accelerate tumorigenesis [15]. Moreover, our previous study found lncRNA CTD-2528L19.6, which was down-regulated during advanced-stage IPF compared to normal lung tissue, alleviate fibroblast activation during the advanced-stage of IPF [12]. Our findings suggest these lncRNAs may be a potential therapeutic target for devastating pulmonary fibrosis.

There are some previous reports of lncRNA regulating the pulmonary fibrosis. Lin et al. found lncRNA Hoxaas3 was up-regulated in the BLM-induced fibrosis in mice, whereas knock-down of lncRNA Hoxaas3 attenuated lung fibrosis [16]. Our previous study revealed lncRNA PFAL, PFAR and PFI was significantly dysregulated in a murine model of pulmonary fibrosis [8, 17, 18]. However, the non-conserved nature of remains to be a great obstacle for the practical applications of lncRNAs. Accordingly, we focused on the DEGs crosstalk analysis of IPF patients and healthy controls to predicting potential target.

Among these candidate key lncRNAs, TP53TG1 was the significant upregulation lncRNA in multiple independent datasets of patient samples as well as the top-ranked lncRNA that had enriched association with the deregulated genes. It has been described that TP53TG1 is shown to function diversely in either oncogenesis or tumor suppression in various tumors. Chen et al. suggested that TP53TG1 is a tumor suppressor, which inhibits growth and metastasis through the WNT/β-catenin signaling pathway in hepatocellular carcinoma [19]. Shao et al. identified that TP53TG1 was an antioncogenic target by survival analysis in breast cancer [20]. In addition, it has been determined that TP53TG1 is an oncogenic lncRNA in nasopharyngeal carcinoma and retinoblastoma [21, 22]. The present study provides the first analysis of the function of TP53TG1 in IPF and fibroblast activation.

LncRNA expression has been described as highly specific to tissue, cell, or disease state, compared to mRNAs [23]. Here, we found that overexpressing TP53TG1 reduced BLM-induced experimental pulmonary fibrosis in mice although TP53TG1 was predicted up-regulated in IPF whole lung. The heterogeneity of lung cell types with distinct expression and epigenetic features may account for the confounding effects. By scRNA-seq analysis, we found the distinct patterns of TP53TG1, which was low expression in fibroblasts cells and high expression in ciliated, ATI cells and basal cells. Further, through systematic screening, we found that TP53TG1 expression was significantly down-regulated in fibroblasts of IPF patients. Fibroblast activation as an important pathophysiological mechanism of pulmonary fibrosis, promotes progressive tissue remodeling and fibrosis [24]. In this study, we found that restoration of TP53TG1 levels in fibroblasts reduced TGF-β1 induced fibrogenesis of human embryonic fibroblast cells and BLM-induced pulmonary fibrosis in experimental mice. In light of these observations, our results suggest that TP53TG1 may serve as a novel target for intervention in fibroblast activation.

Screening for potential interacting proteins for lncRNA is crucial to identify the biological functions of lncRNA. We used ChIRP-MS, an approach that relies upon cross-linking of RNA-protein interactions, significantly improved our knowledge of the TP53TG1-protein interactome and its mechanism of action. Through pathways enrichment analysis of GO and KEGG, it was found that TP53TG1 binding proteins were mainly compact skeleton proteins. Among them, we focused on the up-regulated protein MYH9 in pulmonary fibrosis. Mass spectrometry, RIP, and western blot results showed that TP53TG1 binds to MYH9 and inhibits the expression of MYH9 protein.

The MYH9 gene encodes a heavy chain of non-muscular myosin IIA, which plays a vital role in cell contraction, development, cell morphology, migration, and adhesion [25]. Microarray profiling studies by Kapoun et al. and proteomics of Zhou et al. detected TGF-β1 stimulated increased expression of MYH9 levels in fibroblasts [26, 27]. Liu et al. reported that blebbistatin, an inhibitor of MYH9 ATPase activity, reduced the contractility of activated hepatic stellate cells [28]. Sun et al. found siRNA of MYH9 or blebbistatin significantly impaired cell migration and differentiation, and reduced the contractile capacity of TGF-β1-stimulated fibroblasts containing collagen gels via ALK5/Smad2/3 signaling [29]. These data suggest that MYH9 inhibition can inhibit the differentiation of pulmonary fibroblasts into myofibroblasts. TP53TG1 as a novel MYH9 protein inhibitor to suppress fibroblast activation and pulmonary fibrosis.

Pulmonary fibrosis appears to be the result of interactions between several cell types and cytokines. Our results only confirmed the role and mechanism of TP53TG1 in fibroblasts without systematic interpretation. The distinct expression patterns of TP53TG1 among different cells and their specific functions should be interpreted with caution and examined further in future trials.

**Fig. 8:**
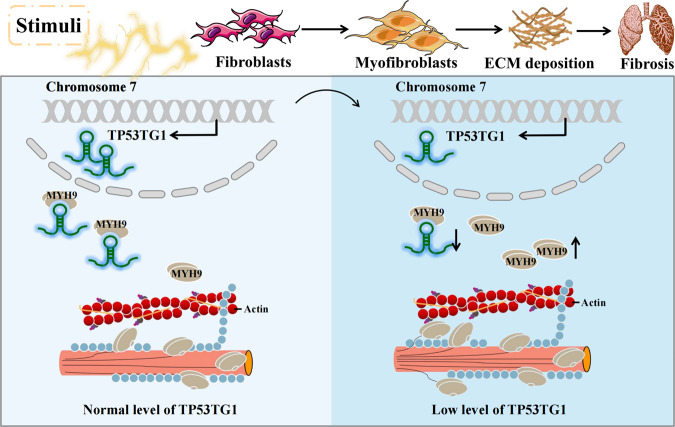
Graphic illustration of TP53TG1 mechanism. TP53TG1 binds to the Myosin Heavy Chain 9 (MYH9) to decrease its protein expression and thus the MYH9-mediated activation of fibroblasts.

Collectively, these data demonstrate the anti-fibrotic role of TP53TG1 in pulmonary fibrosis through inhibiting MYH9 protein expression, which might be attributed to decreased myofibroblasts differentiation and ECM deposition (Fig. 8). TP53TG1 as a MYH9 inhibitor might be target for the control of pulmonary fibrosis.

## Methods

### Data and processing

The RNA sequencing (RNA-seq) profiles for whole lung tissue samples or lung fibroblast samples, scRNA-seq from IPF patients, or control were enrolled from the Gene Expression Omnibus (GEO, http://www.ncbi.nlm.nih.gov/geo/) and Sequence Read Archive (SRA, https://www.ncbi.nlm.nih.gov/sra/) (Table 1).

**Table 1:** Gene expression profiles used in our study.

Tissue	Strategy	Accession	IPF	Control	Platform
Whole lung tissue	RNA-seq	GSE24206	17	6	Affymetrix Human Genome U133
	RNA-seq	SRP108496	18	8	Illumina HiSeq 2500
	RNA-seq	SRP033095	8	7	Illumina HiSeq 2000
	RNA-seq	SRP175341	49	35	Ion Torrent Proton
	scRNA-seq	GSE136831	32	28	Illumina HiSeq 4000
Lung fibroblasts	RNA-seq	GSE44723	10	4	Affymetrix Human Genome U133

If a gene was mapped to multiple probe sets, the expression value for the gene was generated by averaging. Probes that did not map to any Gene ID or map to multiple Gene IDs were deleted.

The RNA-seq profiles were downloaded from SRA. The SRA files were decompressed to fastq files using fasterq-dump (version 2.11.0) [30]. The fastq files were mapped to the human transcriptome based on exon models from GRCh38 using Hisat2 (version 2.2.1) [31]. The resulting SAM files were converted to BAM format using sam tools (version 1.12) [32]. The gene quantification was performed with featureCounts (version 2.0.1) [33].

The profile of 312,928 cells by scRNA-seq has been described by Taylor et al. in GSE136831 [34]. A total of 60 samples, including 32 IPF and 28 normal lungs, were involved in this profile. The quality control, count normalization and correction of mitochondrial influence were performed using Scanpy (version 3.0.1). We removed two cells that had less than expressed 200 genes and filtered out 1987 genes detected in less than 3 cells. 4798 single cells with more than 5000 genes or with more than 30% mitochondrion-derived unique molecular identifier (UMI) counts were considered as low-quality cells and removed. All single cells were annotated into 39 types by gene markers.

### Identification of IPF related genes

We detected differentially expressed genes using Student’s *t*-test with *P*-value <0.01. The differentially expressed genes were defined as IPF related lncRNAs or IPF related mRNAs, as shown in Supplementary Fig. 1.

### Co-expression network construction

The Pearson correlation test was used to test correlations between differential expressed lncRNAs and mRNAs. The lncRNA-mRNA pairs with an absolute value of correlation coefficient *r* > 0.8 and *P*-value < 0.01 were included in the co-expression network. The methodological workflow is summarized in Supplementary Fig. 1.

### Fibrosis related gene sets

Fibrosis-related gene sets were collected from the Molecular Signatures Database (MSigDB, https://www.gsea-msigdb.org/gsea/msigdb), including fibroblast-related gene sets. Fibrosis-related gene sets include fibrosis-related biochemical pathways, signaling cascades, expression profiles from publications and other biological concepts [35].

### Protein-protein interactions (PPI) and transcriptional regulation interactions

PPI was collected from the Pathway Commons database v12 (http://www.pathwaycommons.org/). Cytoscape software version 3.6.0 (https://cytoscape.org/) was used to visualize the interaction network. Transcriptional regulation interactions were obtained by integrating multiple databases (ChIPBase, http://rna.sysu.edu.cn/chipbase/; MsigDB, https://www.gsea-msigdb.org/gsea/msigdb; TRANSFAC, https://genexplain.com/transfac/).

### Pathway enrichment analysis

Functional analysis was performed to annotate functions on given genes based on the six top categories (09100 to 09160) from the Kyoto Encyclopedia of Genes and Genomes (KEGG, http://www.genome.jp/kegg/). The hypergeometric distribution model was used to test whether pathways were enriched with genes in the co-expression network with the method “enrichKEGG” in R package clusterProfiler (version 4.0.5). The *P*-value was adjusted by the Benjamini-Hochberg procedure. The pathways with false discovery rate (FDR) less than 0.05 were considered to be significant. Disease-specific pathways were excluded from the functional analysis.

### Mouse pulmonary fibrosis models

Six weeks old C57BL/6 male mice at average weight 18–22 g. Animals were randomly assigned and anesthetized by intraperitoneal injection of Avertin (250 mg/Kg). To induce pulmonary fibrosis, mice were intratracheally injected with BLM at 3 mg/Kg or saline and sacrificed 21 days after BLM injection. All animal experiments were approved by the Harbin Medical University College of Pharmacy Ethical committee (NO. IRB300720) and complied with the Helsinki Declarations. Assessors were un-blinded to group allocation. No statistical methods were used to pre-determine sample sizes but our sample sizes are greater than or equal to 3 for each condition.

### Micro-CT scanning

After mice intratracheal instillation of BLM 21 days, CT scanning was used to measure lung density and the degree of pulmonary fibrosis. Mice were anesthetized with isoflurane gas. Then mice were lying in the supine position and gently immobilized with tape. The following scan parameters were set: tube voltage, 120 kV; exposure time, 0.5 s; scanning thickness, 0.5 mm. For a total scan time of 3 min per mouse. CT images were analyzed by the relevant software of the system.

### Hydroxyproline assay

To detect the hydroxyproline contents, lung tissues were processed according to the hydroxyproline assay kit instructions (Nanjing Jiancheng Bio Co., Cat No. A030-2-1, Nanjing, China). Lung tissues were weighed 45 mg wet weight and incubated with 1 mL of hydrolysis solution, then hydrolyzed in boiling water bath for 20 min. Afterward, mixed the pH solution until the liquid color changed to yellow-green and added 10 mL double-distilled water. Then mixed hydrolysis solution with an appropriate amount of activated carbon, centrifuge at 3500 rpm for 10 mins. Each sample aspirated the supernatant to determine the absorbance at 550 nm, and calculated the content of hydroxyproline in the tissue according to the formula.

### Immunohistochemistry

Fresh lung tissues were fixed with 4% paraformaldehyde for 48 h and then dehydrated, embedded, and sectioned in paraffin. 5 μm lung sections were stained with H&E (G1120, Solarbio, China) and Masson’s trichrome staining kit (G1340, Solarbio, China). The steps of immunohistochemical staining were as follows: lung sections were dewaxed transparently and treated with 3% H_2_O_2_ dropwise for 10 mins; distilled wash with water and immerse slides in 0.01% sodium citrate solution, microwave to boiling, 50% goat serum blocked 30 mins, overnight incubation with primary antibody α-SMA (1:200, AF1032, Affinity) and Collagen I (1:200, AF7001, Affinity). The next day incubated horseradish peroxidase-labeled antibody for 30 min at 37 °C and developed with DAB chromogen. The nuclei were stained with hematoxylin for 5 min, and the sections were rinsed with running water for 10 min. After being sealed with neutral balsam, five visual fields were randomly selected for evaluation in each section.

### Cell culture and transfection

The human embryonic lung fibroblasts MRC-5 (authenticated by STR identification) was purchased from Cell Bank of Chinese Academy of Sciences, and cultured in MEM medium supplemented with 10% fetal bovine serum (FBS) containing Gluta-max dipeptide 1 mL, sodium pyruvate solution 1 mL, 100 U/mL penicillin-streptomycin Gray, then plated in 5% CO_2_ at 37 °C air incubator. Primary mouse lung fibroblasts (PMLFs) were isolated and as previously described [18]. TP53TG1 plasmid (Biowit Technologies, Shenzhen, China) and smart silencer (RiboBio, Guangzhou, China) were transfected using Lipo2000 according to the manufacturer’s instructions. TGF-β1 (Sigma–Aldrich, U.S.A.) was used to induce fibrogenesis at 10 ng/mL. After being cultured with TGF-β1 for 48 h, the cells were prepared for further analysis.

### Fluorescence in situ hybridization

To determine the cellular localization of lncRNA TP53TG1 in MRC-5, fluorescence in situ hybridization (FISH) was performed using the lncRNA FISH Probe Mix kit (Ribobio, Guangzhou, China). Briefly, cells were fixed in 4% paraformaldehyde (Solarbio, China) for 10 min at room temperature and then incubated in 1 mL pre-cooled permeable solution for 5 min at 4 °C. Then the cells were incubated with 200 μL pre-hybridization buffer for 30 min. Then the cells hybridized with the hybridization buffer containing lncRNA FISH Probe Mix at 37°C overnight. The next day cells were washed separately with 4 × SSC, 2 × SSC, and 1 × SSC at 42 °C for 5 min. Nuclei staining with DAPI (Roche, Basel, Switzerland). The images were taken under the inverted fluorescence microscope (Olympus, IX73, Japan).

### Quantitative real-time PCR (qRT-PCR)

The total RNA of cells was extracted with TRIzol reagent. The concentration and purity of extracted RNA were determined by NanoDrop 8000 (Thermo, U.S.A). RNA reverse transcription for complementary DNA (cDNA) by using 5x All-in-One RT Master Mix. cDNA was used to detect the relative mRNA level by qRT-PCR in the presence of SYBR Green fluorescent dye (Applied Biosystems, Foster City, CA). The relative mRNA level was calculated by the 2^-ΔΔCt^ method based on the Ct values and normalized to the GAPDH expression of each sample.

### Western blot

Total proteins of cells or tissues were extracted and lysed with RIPA buffer (Beyotime Jiangsu, China) containing protease inhibitor. The protein samples were separated on 10% SDS-polyacrylamide gel electrophoresis and transferred to pure nitrocellulose (Pall Life Sciences, Ann Arbor, MI, U.S.A.). The membranes were hybridized with β-actin (1:2000, Proteintech, 20536-1-AP), Fn1 (1:500, Proteintech, 15613-1-AP), Collagen I (1:500, Wanlei, WL0088) and α-SMA (1:1000, Affinity, AF1032) primary antibodies at 4°C overnight. The Odyssey Infrared Imaging System (Odyssey CLX, Biosciences, U.S.A.) was used to detect the protein expression and quantitatively analyzed by Image Studio Ver 5.2 software.

### Immunofluorescence staining

After cells were transfected and treated with TGF-β1 for 48 h and washed by PBS three times, then fixed with 4% paraformaldehyde for 30 mins. The fixed cells were permeabilized with 0.1% penetration buffer for 1 h. Later the cells were blocked with 50% goat serum at 37 °C for 1 h. Cells were incubated with α-SMA antibody (1:200, Abcam, ab7817) at 4 °C overnight. After incubation in anti-mouse IgG (H + L) (1:200, Alexa Fluor 594, #8890, Cell Signaling Technology) at room temperature for 1 h. Nuclei staining was carried out with DAPI for 5 mins. The cells were photographed under a fluorescence microscope.

### EdU cell proliferation assay

The MRC-5/PMLFs proliferation was detected by the Cell-Light EdU DNA cell proliferation kit (RiboBio, Guangzhou, China). The cells were added with 200 μL, 50 μM EdU solution and incubated for 2 h. Then cells were fixed with 4% paraformaldehyde at room temperature for 30 min and incubated in 2 mg/mL glycine solution to neutralize paraformaldehyde for 5 min. Afterward, cells were stained with Apollo Dye solution for proliferating cells and the Nuclei was stained by DAPI. Images photographed under the inverted fluorescence microscope.

### Wound-healing migration assay

MRC-5/PMLFs were seeded into 6-well plates. When the cells grew to a monolayer, the cells were gently scratched with a 10 μL pipette tip. The scratch healing areas were observed and photographed under a microscope. Afterward, the cells were transfected and added TGF-β1. The images were taken by the Nikon Ts100 microscope (Nikon, Tokyo, Japan) at 0, 24 h. The images of cells migration were analyzed by using Image J.

### ChIRP-MS

The complex formed by RNA, protein, and nucleic acid in MRC-5 cells was fixed by formaldehyde cross-linking, and then the bound protein and nucleic acid were purified by biotin-labeled lncRNA TP53TG1 reverse complementary probe and streptomycin magnetic beads. Afterward, the proteins were digested by trypsin into peptides and then analyzed by the mass spectrometer and identified by mass spectrum peaks (Kangcheng Bio-tech, Shanghai, China).

### RNA binding protein immunoprecipitation (RIP) assay

RIP assays were performed according to the manufacturer’s instructions of the Magna RIP Kit (#17-700, Millipore, CA, USA). LncRNA TP53TG1 bound proteins were analyzed by western blot. The co-precipitated RNAs were detected by qRT-PCR.

### Statistics and analysis

All public datasets statistical analyses in this study were carried out using R software version 3.6.0 (http://www.r-project.org/). The statistical tests were justified as appropriate. Variance is similar between comparison groups. All experimental data were presented as mean ± SEM. The Student’s *t*-test was used to detect differentially expressed genes between two groups. Multiple comparisons were used one-way analysis of variance (ANOVA), and Bonferroni corrections were used for multiple comparisons. *P* < 0.05 was considered a statistically significant difference. Experimental statistical analyses were performed with GraphPad Prism 8.0.

## Supplementary information

Supplementary Figures

Original data of western blot

checklist

## Data Availability

All data that support the findings of this study are available from the corresponding author upon reasonable request.
