# Supplementary material for: Systematic analyses identify the anti-fibrotic role of lncRNA TP53TG1 in IPF
Source: Cell Death Dis. 2022 Jun 4;13(6):525. doi: 10.1038/s41419-022-04975-7 (PMC9166247; doi:10.1038/s41419-022-04975-7)
Supplement: Supplementary file 1 — Supplementary Figures [file 41419_2022_4975_MOESM1_ESM.docx]

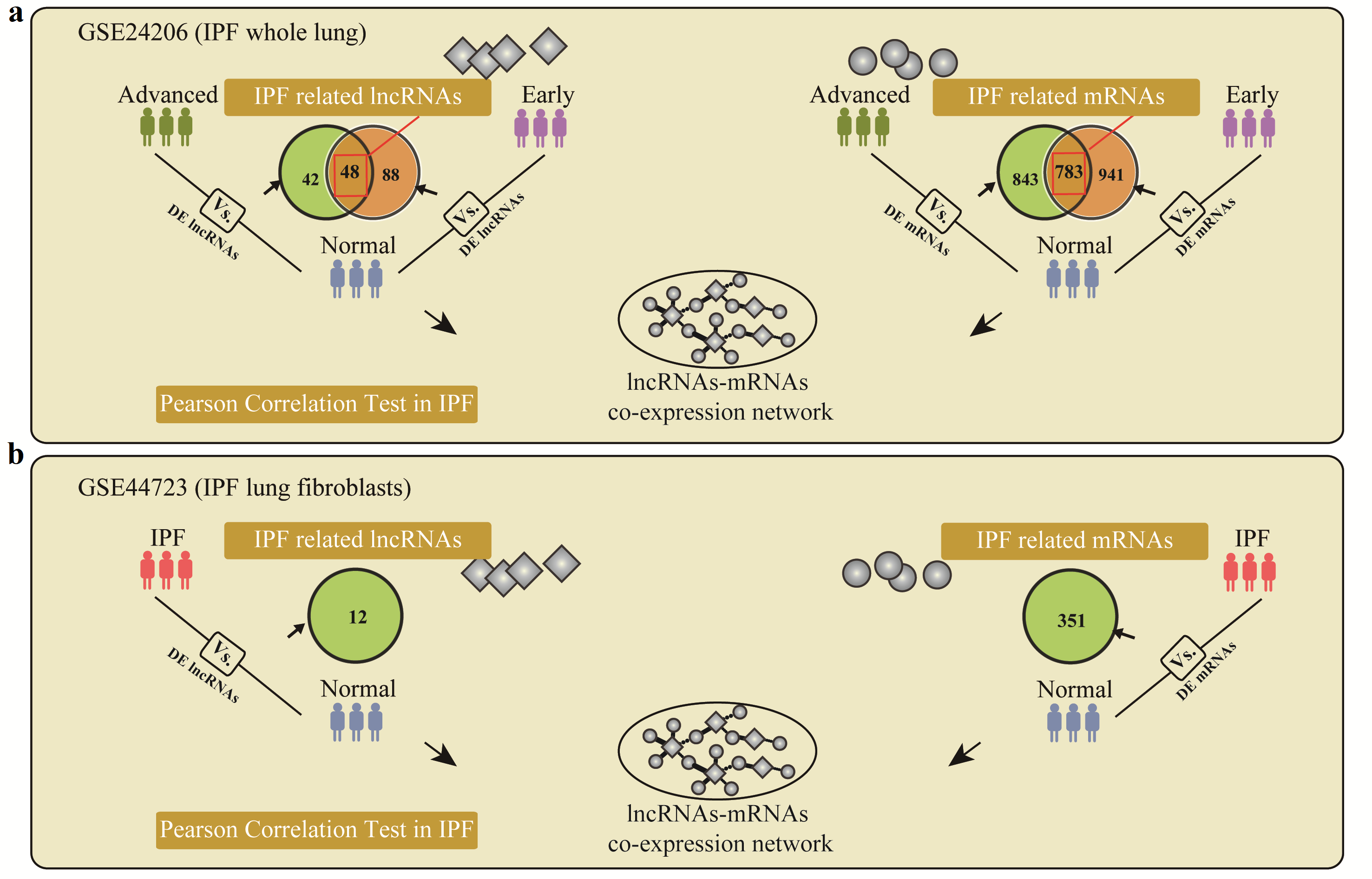


**Supplementary Fig. 1 Identification of IPF related genes.** (a) Venn diagram of overlapping differentially expressed genes (mRNAs and lncRNAs) among two groups of comparison: early IPF vs. normal lung, advanced IPF vs. normal lung in whole lung samples from GSE24206. Correlations between IPF related mRNAs and lncRNAs were calculated to construct the IPF co-expression network (|r| >0.8, *P*-value < 0.01, Pearson Correlation Test). (b) Differentially expressed genes (mRNAs and lncRNAs) between IPF and normal lung in lung fibroblasts from GSE44723. Correlations between IPF related mRNAs and lncRNAs were calculated to construct the IPF co-expression network (|r| >0.8, *P*-value < 0.01, Pearson Correlation Test). DE, differentially expressed.


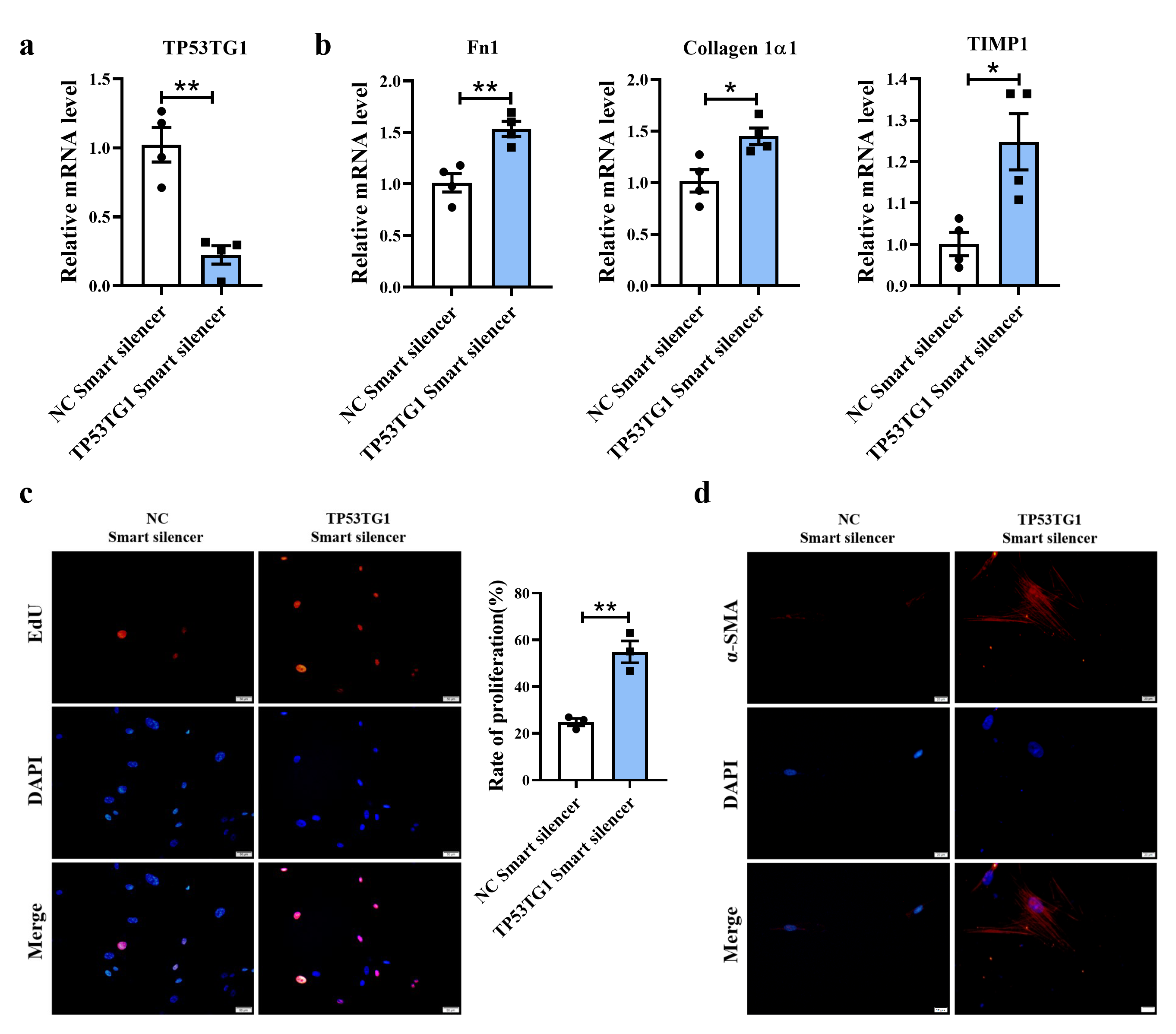


**Supplementary Fig. 2 Effects of TP53TG1 knockdown on MRC-5 activation.** (a) The knockdown efficiency of TP53TG1 smart silencer evaluated by qRT-PCR assay. (b) Knockdown of TP53TG1 induced significant variations of Fn1, Collagen 1α1, TIMP1 mRNA level. (c, d) EdU assay and immunofluorescent staining showed that TP53TG1 knockdown induced proliferation(bar=50 μm, n=3) and differentiation (bar=20 μm; n=3) of MRC-5 cells. **P* < 0.05, ***P* < 0.01.


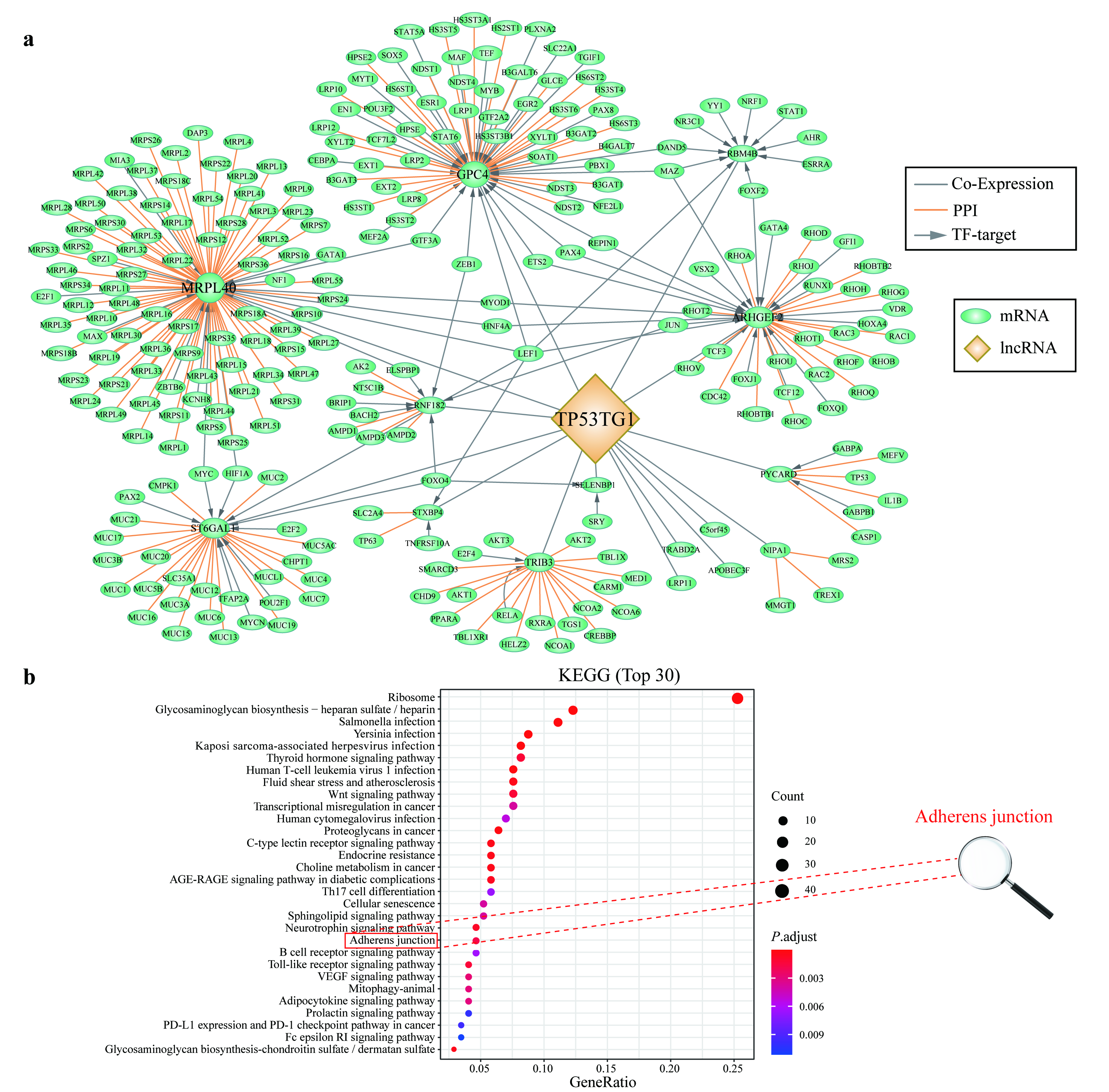


**Supplementary Fig. 3 TP53TG1 interaction network.** (a) TP53TG1 (yellow oval) was linked to other genes (green oval) through co-expression (black edges), transcriptional regulation (black edges with arrows) or PPI (blue edges). (b) KEGG pathways enrichment with all TP53TG1 related genes in the interaction network (top 30). FDR < 0.05, Hypergeometric test. The size of bubble indicates the number of genes in the corresponding annotation. The color shade corresponds to the -log10 (P-value) value. The closer the color is to red, the more significant the enrichment is.
