# Supplementary material for: Systematic analyses identify the anti-fibrotic role of lncRNA TP53TG1 in IPF
Source: Cell Death Dis. 2022 Jun 4;13(6):525. doi: 10.1038/s41419-022-04975-7 (PMC9166247; doi:10.1038/s41419-022-04975-7)

Figure2D (n=3)

Prevention

20211012

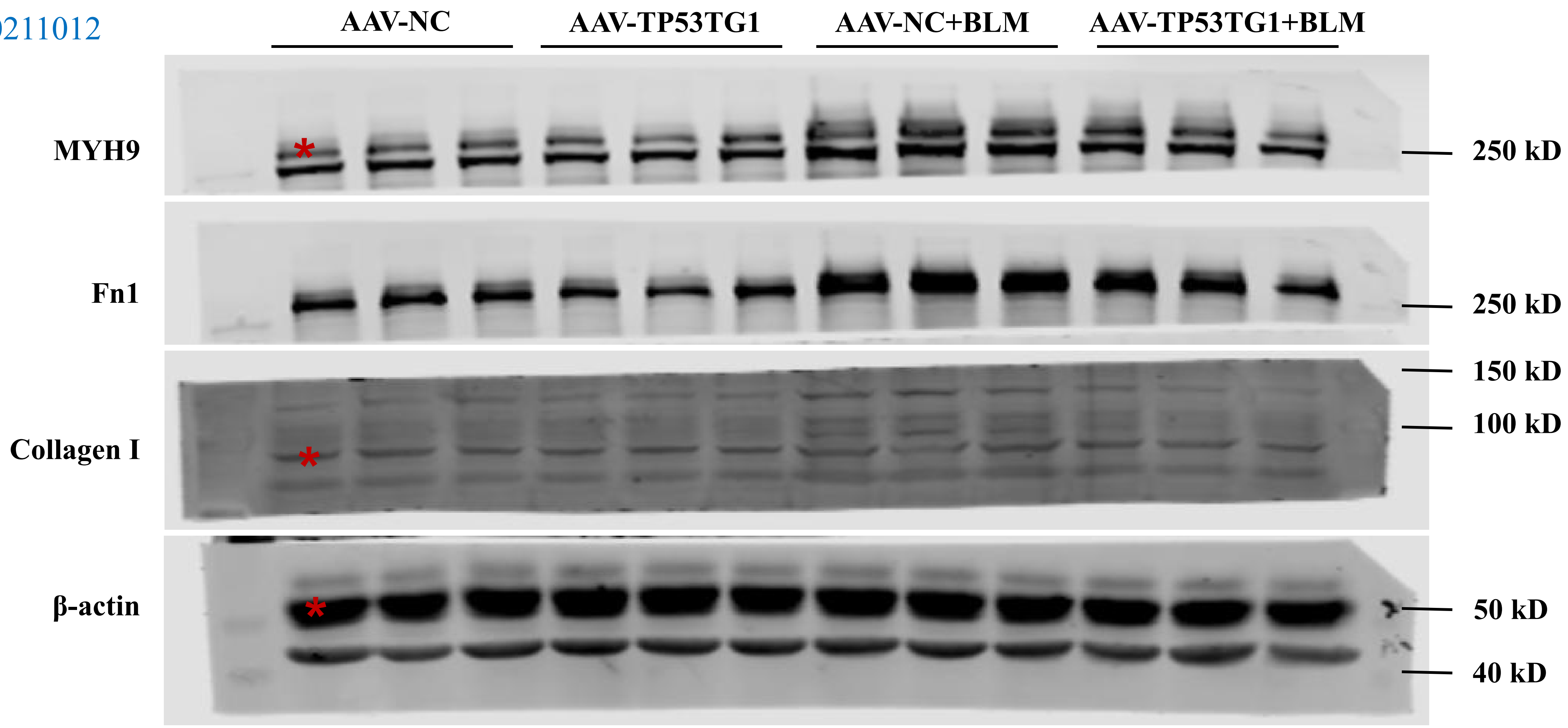

Figure 6C (n=3)

Therapy

20211008

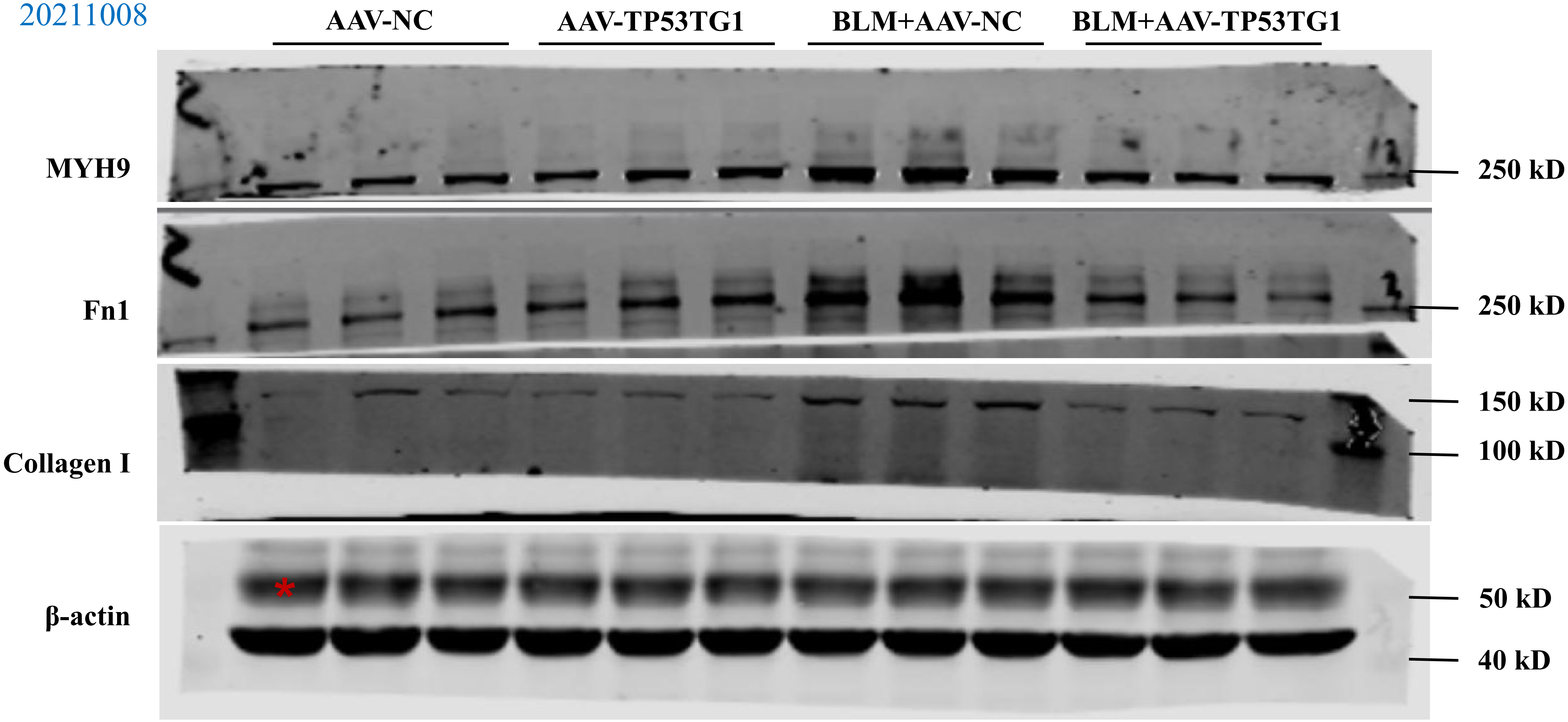

Figure 7J-7K (n=4)

20211020

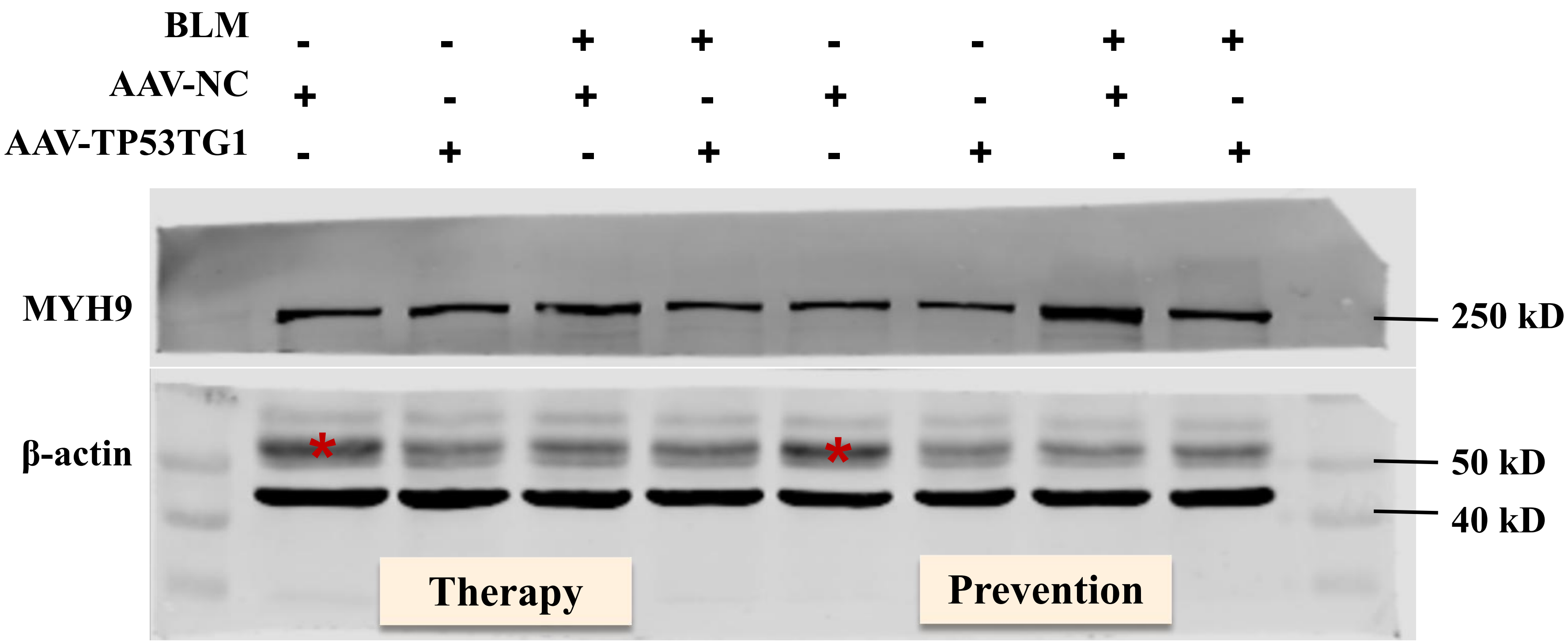

MRC-5 Figure4D (n=4)

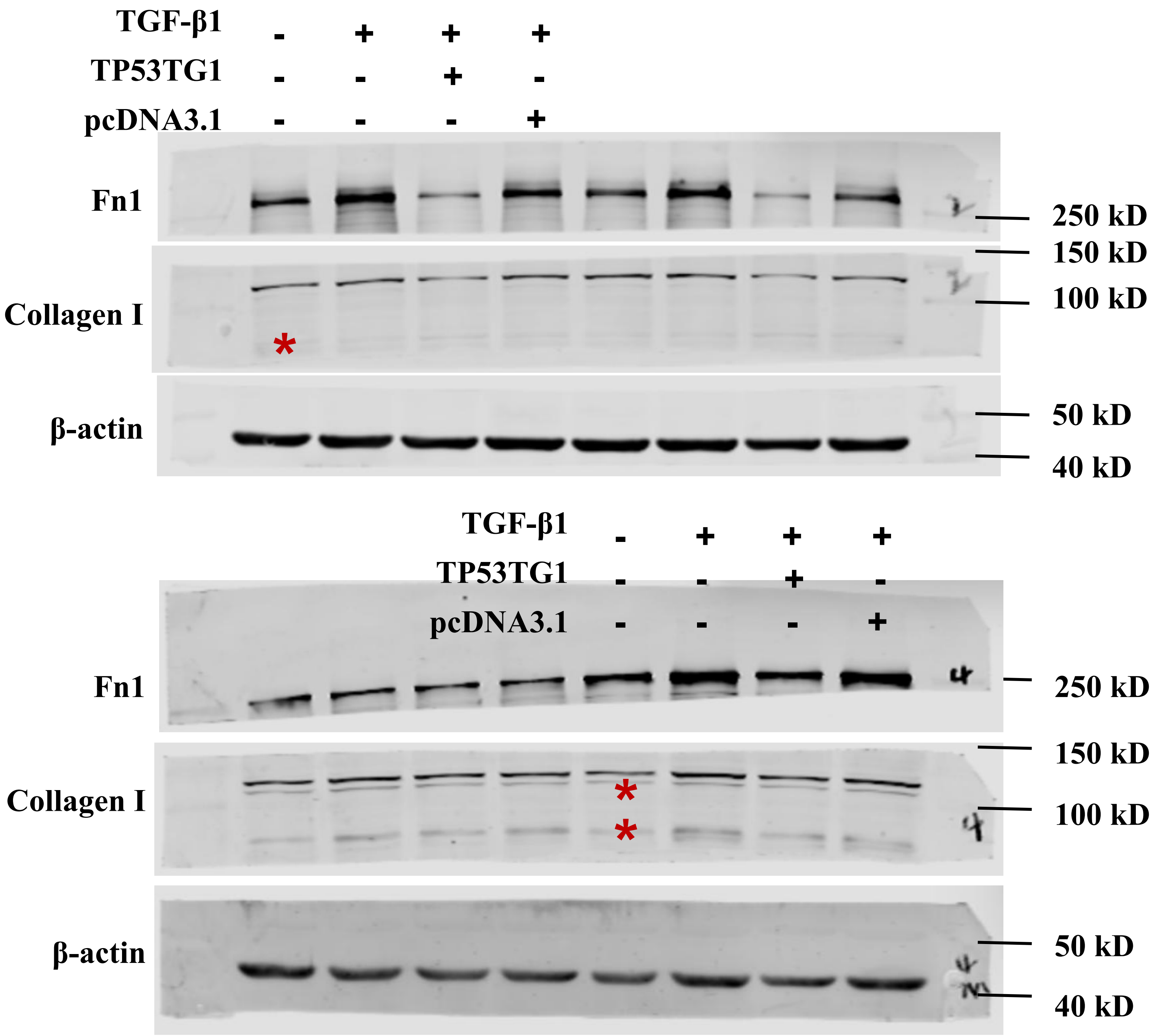

MRC-5 Figure4D (n=4)

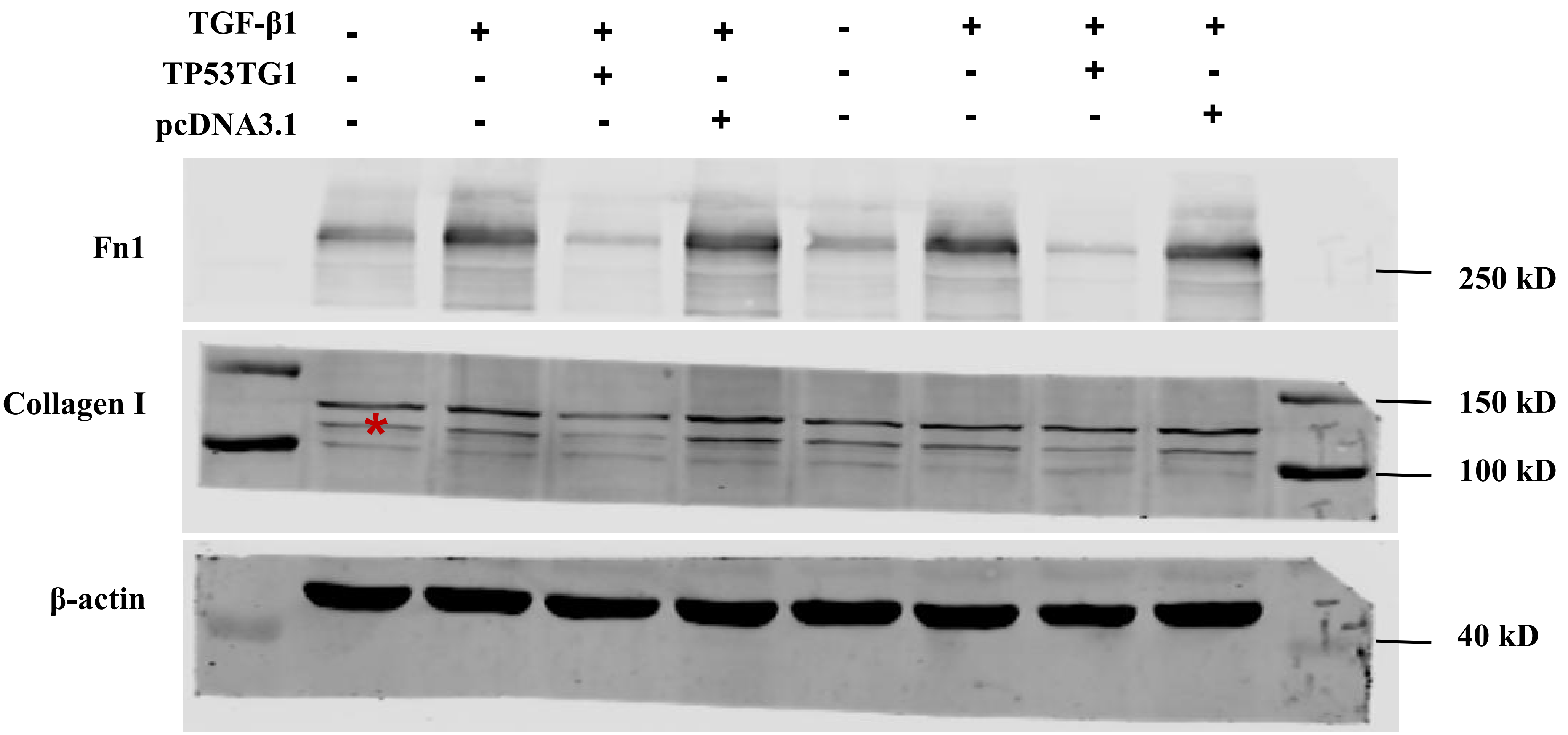

PMLFS Figure5B (n=5)

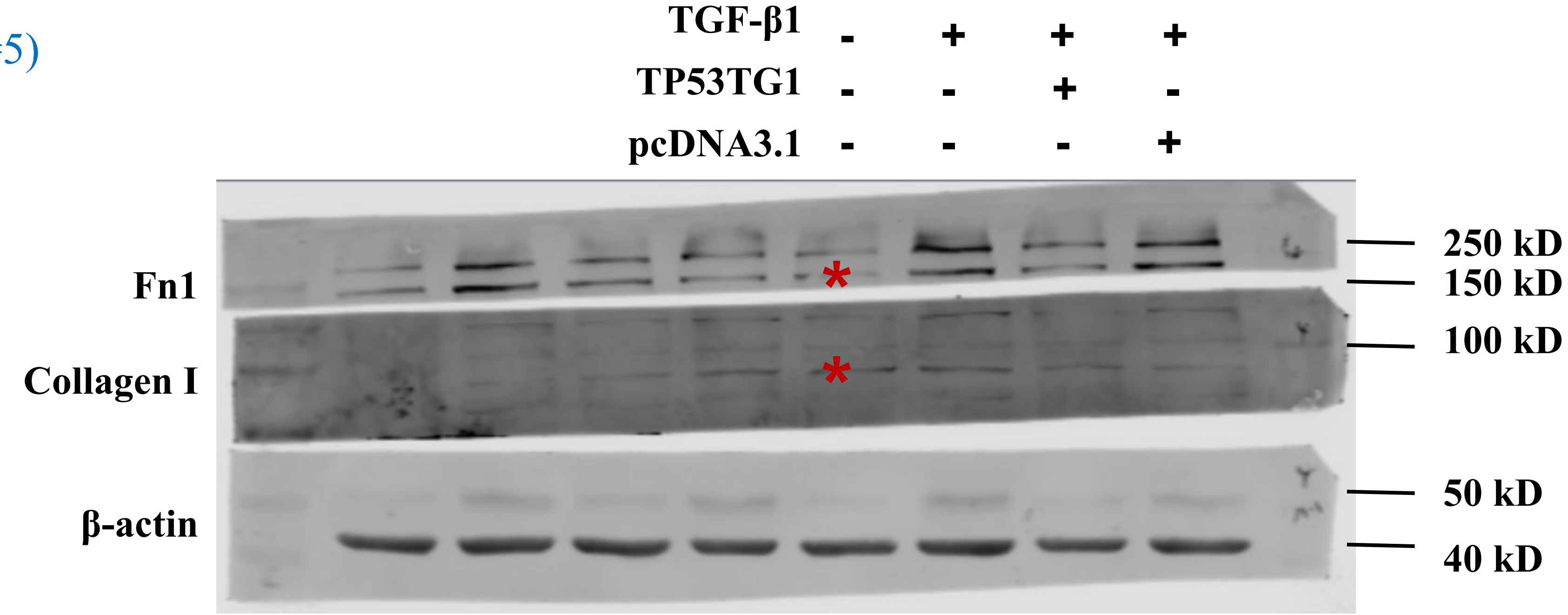

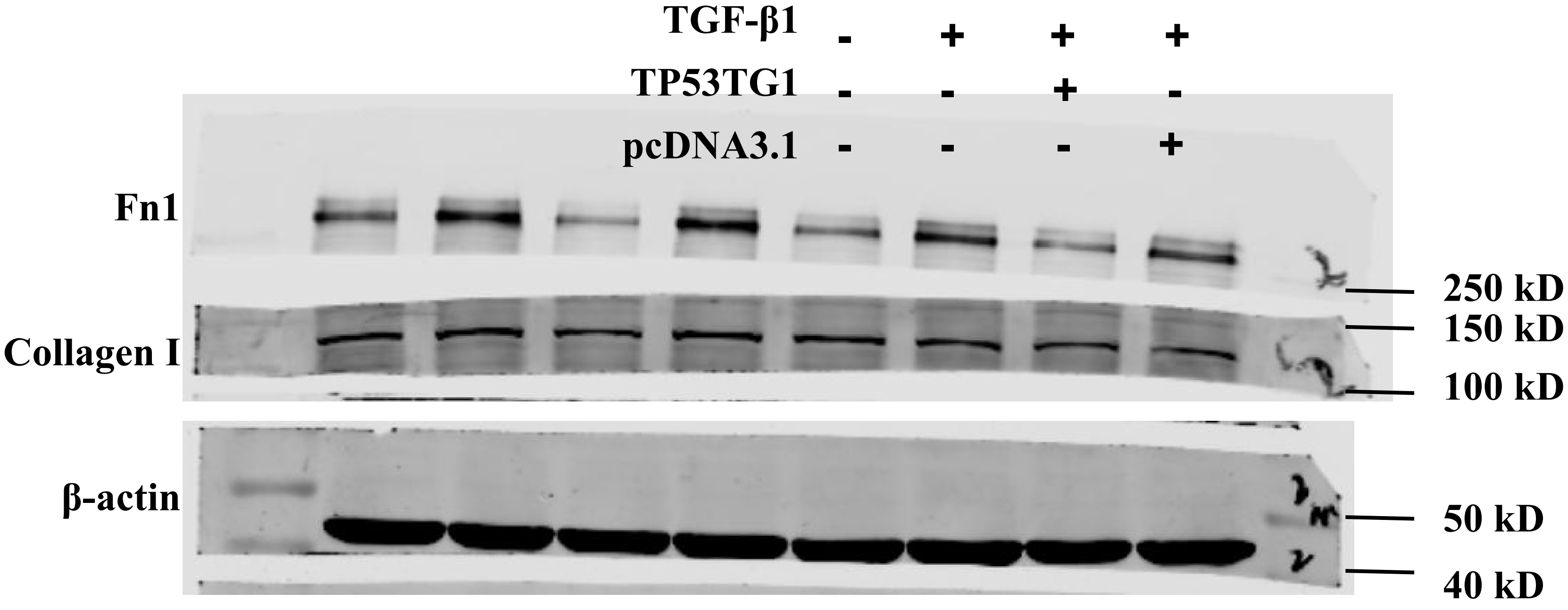

PMLFS Figure5B (n=5)

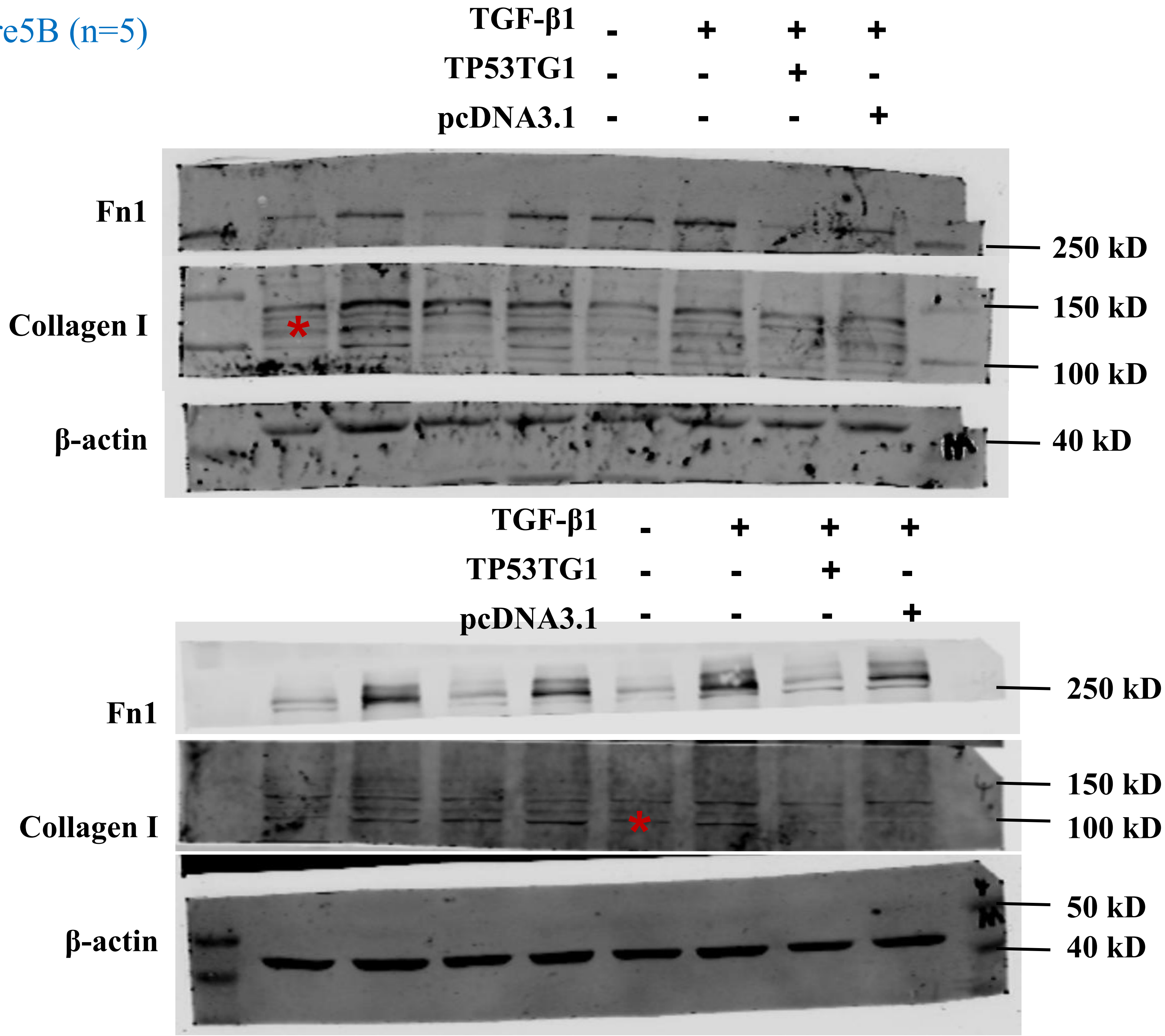

PMLFS Figure5B (n=5)

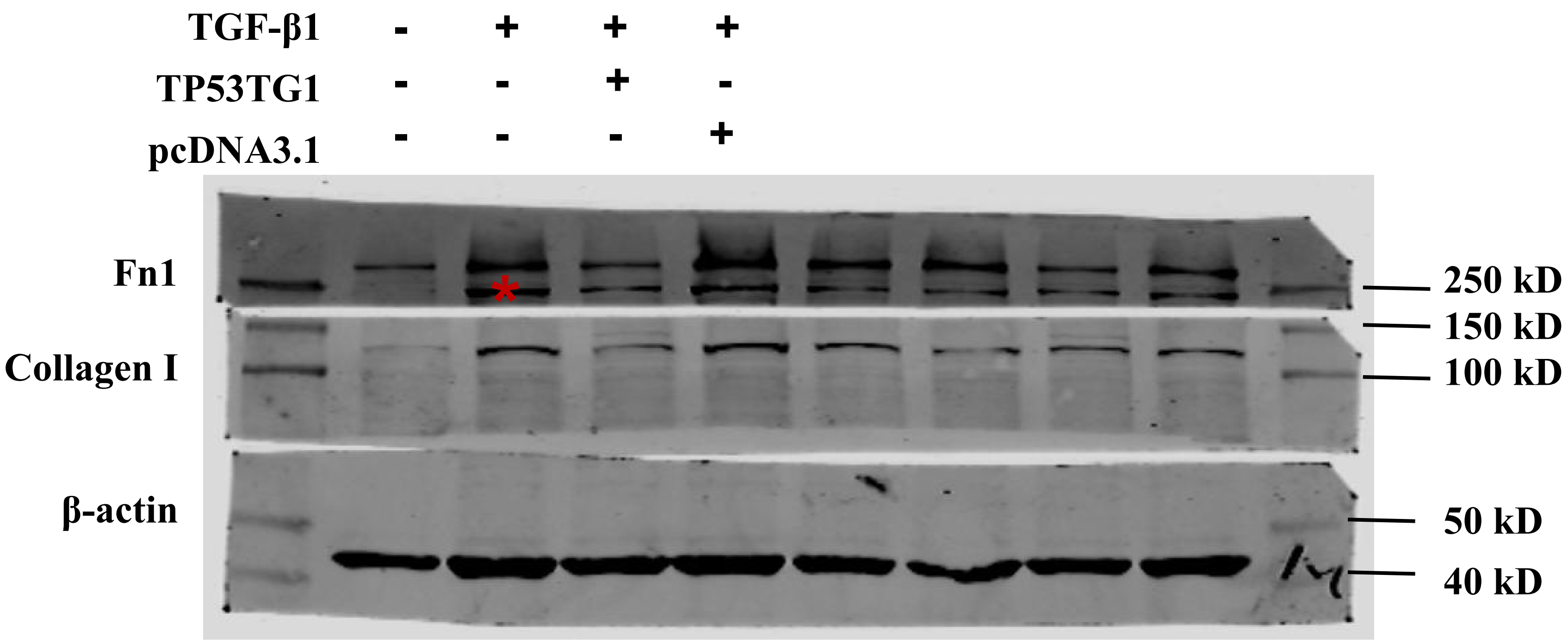

MRC-5 Figure 7H (n=4)

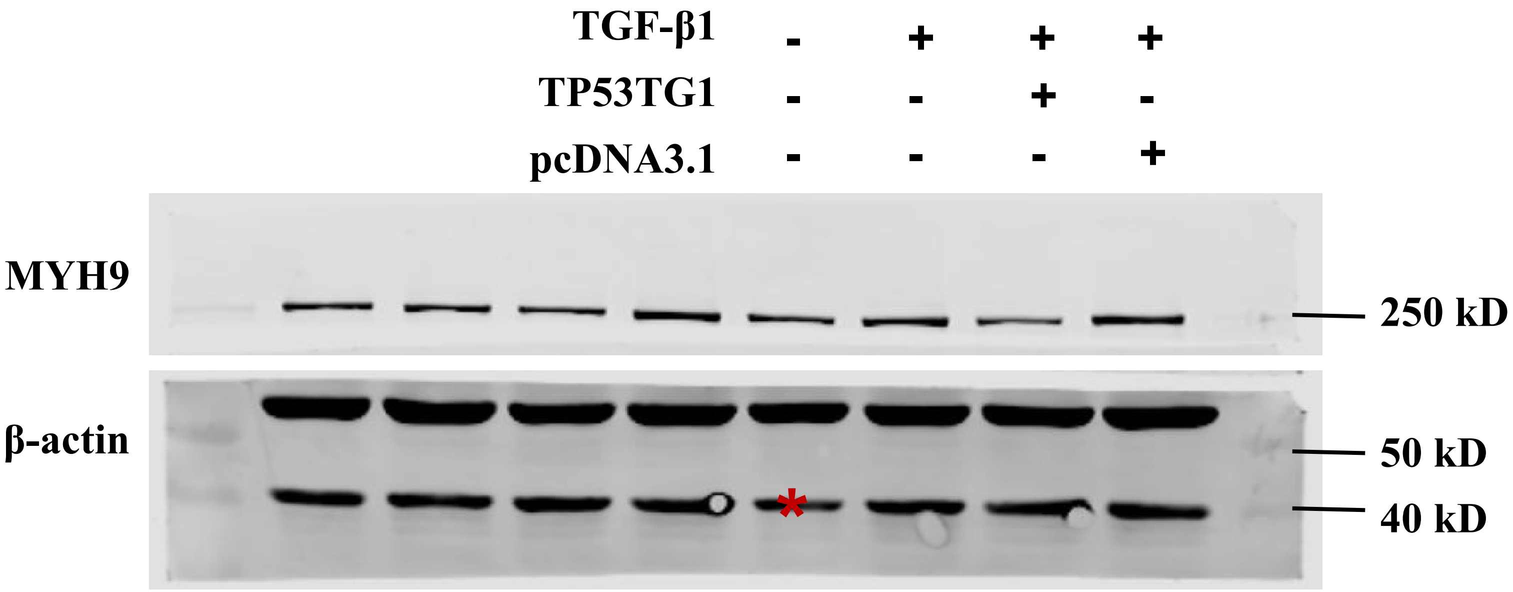

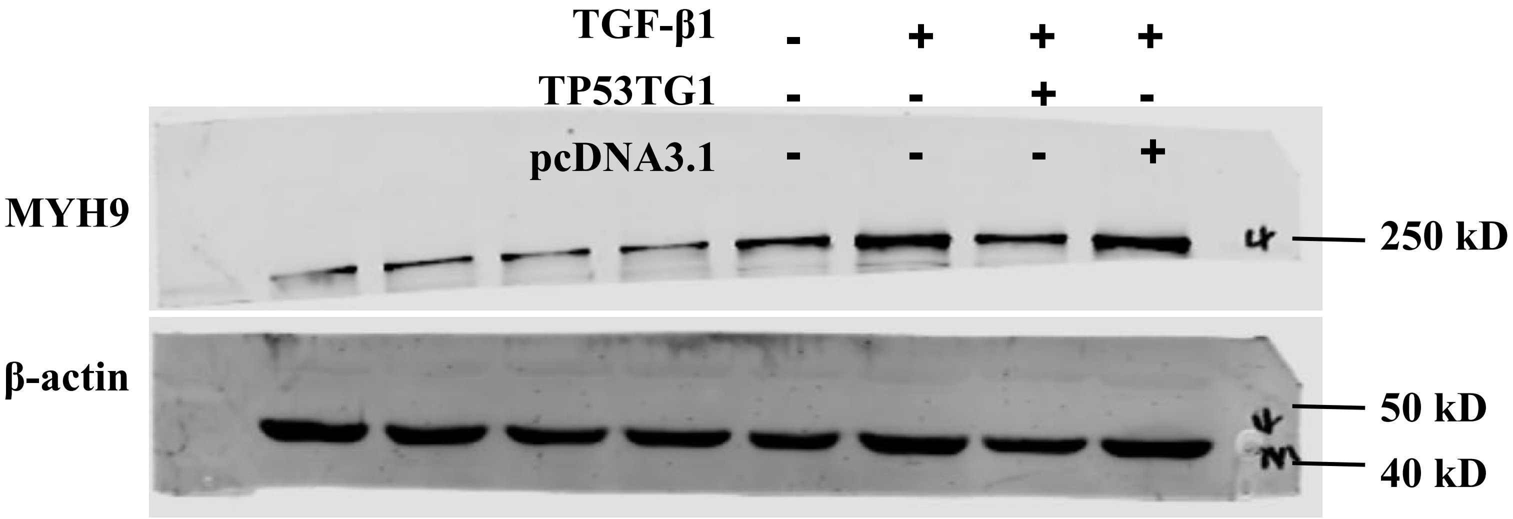

MRC-5 Figure 7H (n=4)

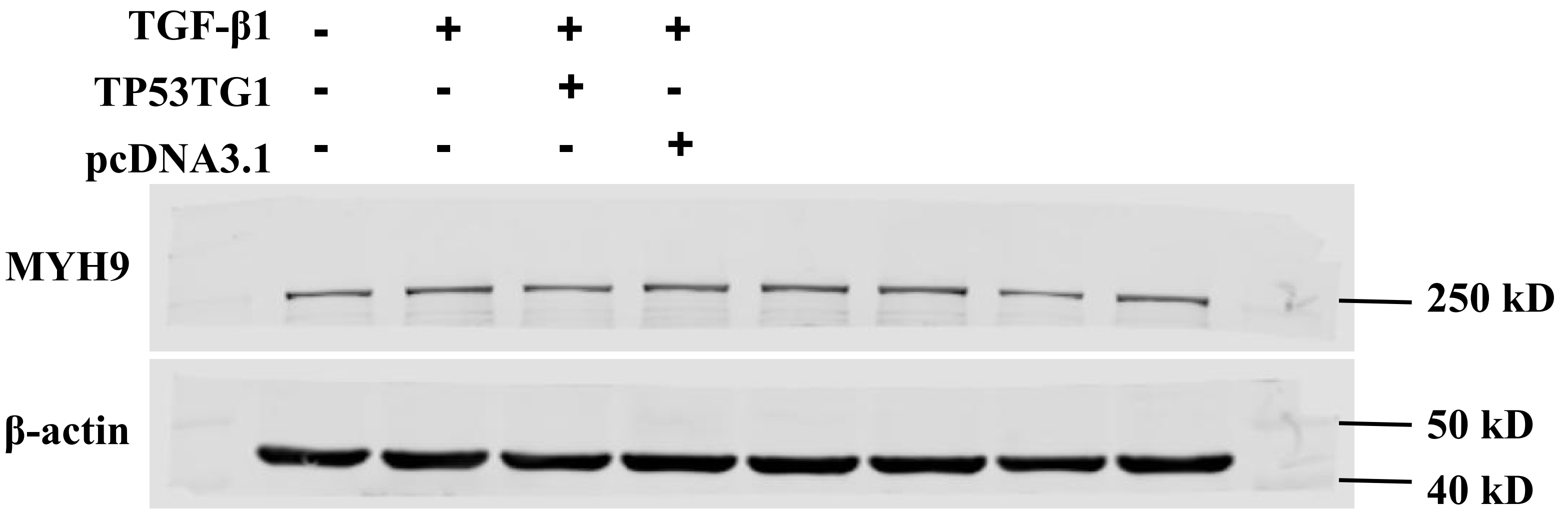

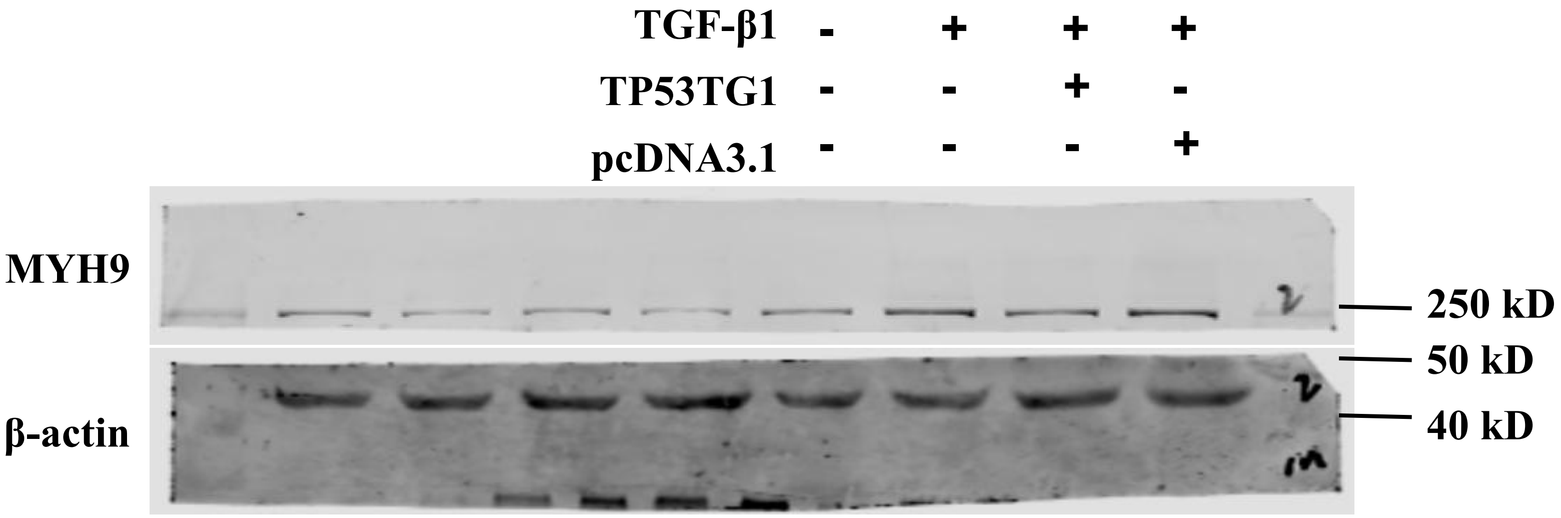

PMLFS Figure 7I (n=4)

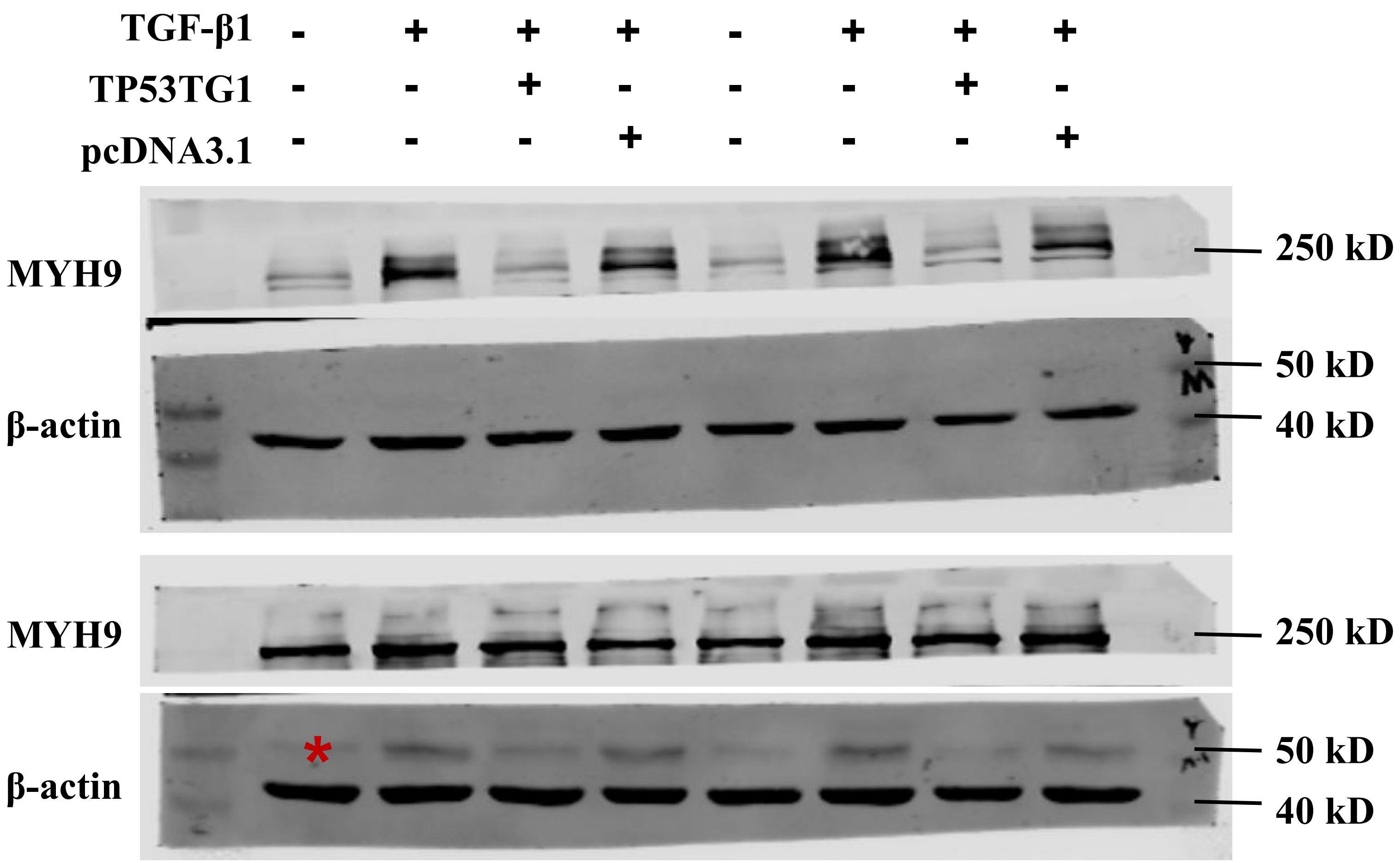

Supplement: Supplementary file 2 — Original data of western blot [file 41419_2022_4975_MOESM2_ESM.pdf]
